# Supplementary material for: Effects of Consuming Ounce-Equivalent Portions of Animal- vs. Plant-Based Protein Foods, as Defined by the Dietary Guidelines for Americans on Essential Amino Acids Bioavailability in Young and Older Adults: Two Cross-Over Randomized Controlled Trials
Source: Nutrients. 2023 Jun 25;15(13):2870. doi: 10.3390/nu15132870 (PMC10343739; doi:10.3390/nu15132870)
Supplement: Supplementary file 1 [file nutrients-15-02870-s001.zip › Supplementary Figures.pdf]

Effects of consuming ounce equivalent portions of animal- vs. plant-based Protein Foods, as defined by the Dietary Guidelines for Americans on essential amino acids bioavailability in young and older adults: crossover randomized controlled trials. Gavin Connolly

List of Supplemental Figures

Figure S1. Plasma total amino acids concentrations for young adults (Figure S1A) and older adults (Figure S1B) in the fasted (0-minute) and postprandial (300-minute) period after initiation of meal consumption for pork, eggs, black beans, and almonds. Different symbols indicate a significant difference between or among trials at each time point (Bonferroni adjusted, $P < 0.05$). * denotes a significant difference between pork or eggs vs. black beans. † denotes a significant difference between pork or eggs vs almonds. ‡ denotes a significant difference between pork vs eggs. ¥ denotes significant difference between pork vs. almonds. # denotes a significant difference between pork vs. black beans. Figure S1C. Plasma total amino acids positive incremental area under the curve (iAUCpos) for pork (black bars), eggs (gray bars), black beans (white bars), and almonds (dashed black and white bars) for young and older adults. Different letters indicate a significant difference among trials within age group (Bonferroni adjusted $P < 0.05$). All values are least squares means ± SE; young adults: n = 30 (15 females, 15 males); older adults: n = 25 (15 females, 10 males).

Figure S2. Plasma branched-chain amino acids concentrations for young adults (Figure S2A) and older adults (Figure S2B) in the fasted (0-minute) and postprandial (300-minute) period after initiation of meal consumption for pork, eggs, black beans, and almonds. Different symbols indicate a significant difference between or among trials at each time point (Bonferroni adjusted, $P < 0.05$). * denotes a significant difference between pork or eggs vs. black beans. † denotes a significant difference between pork or eggs vs almonds. ‡ denotes a significant difference

between pork vs eggs. ¥ denotes significant difference between pork vs. almonds. Figure S2C. Plasma branched-chain amino acids positive incremental area under the curve (iAUCpos) for pork (black bars), eggs (gray bars), black beans (white bars), and almonds (dashed black and white bars) for young and older adults. Different letters indicate a significant difference among trials within age group (Bonferroni adjusted $P < 0.05$). All values are least squares means ± SE; young adults: n = 30 (15 females, 15 males); older adults: n = 25 (15 females, 10 males).

Figure S3. Plasma leucine concentrations for young adults (Figure S3A) and older adults (Figure S3B) in the fasted (0-minute) and postprandial (300-minute) period after initiation of meal consumption for pork, eggs, black beans, and almonds. Different symbols indicate a significant difference between or among trials at each time point (Bonferroni adjusted, $P < 0.05$). * denotes a significant difference between pork or eggs vs. black beans. † denotes a significant difference between pork or eggs vs almonds. ‡ denotes a significant difference between pork vs eggs. ¥ denotes significant difference between pork vs. almonds. # denotes a significant difference between pork vs. black beans. Figure S3C. Plasma leucine positive incremental area under the curve (iAUCpos) for pork (black bars), eggs (gray bars), black beans (white bars), and almonds (dashed black and white bars) for young and older adults. Different letters indicate a significant difference among trials within age group (Bonferroni adjusted $P < 0.05$). * denotes a significant difference between young and older adults for pork (Bonferroni adjusted $P < 0.05$). All values are least squares means ± SE; young adults: n = 30 (15 females, 15 males); older adults: n = 25 (15 females, 10 males).

Effects of consuming ounce equivalent portions of animal- vs. plant-based Protein Foods, as defined by the Dietary Guidelines for Americans on essential amino acids bioavailability in young and older adults: crossover randomized controlled trials. Gavin Connolly

Figure S4. Serum glucose concentrations for young adults (Figure S4A) and older adults (Figure S4B) in the fasted (0-minute) and postprandial (300-minute) period after initiation of meal consumption for pork, eggs, black beans, and almonds. Different symbols indicate a significant difference between or among trials at each time point (Bonferroni adjusted, $P < 0.05$). $ denotes a significant difference between eggs vs. black beans. £ denotes a significant difference between eggs vs. almonds. Figure S4C. Serum glucose positive incremental area under the curve (iAUCpos) for pork (black bars), eggs (gray bars), black beans (white bars), and almonds (dashed black and white bars) for young and older adults. * denotes a significant difference between young and older adults for black beans (Bonferroni adjusted $P < 0.05$). All values are least squares means ± SE; young adults: n = 30 (15 females, 15 males); older adults: n = 25 (15 females, 10 males).

Figure S5. Serum insulin concentrations for young adults (Figure S5A) and older adults (Figure S5B) in the fasted (0-minute) and postprandial (300-minute) period after initiation of meal consumption for pork, eggs, black beans, and almonds. Different symbols indicate a significant difference between or among trials at each time point (Bonferroni adjusted, $P < 0.05$). $ denotes a significant difference between eggs vs. black beans. ¥ denotes a significant difference between pork vs. almonds. ¢ denotes a significant between black beans vs. almonds. Figure S5C. Serum insulin positive incremental area under the curve (iAUCpos) for pork (black bars), eggs (gray bars), black beans (white bars), and almonds (dashed black and white bars) for young and older

adults. All values are least squares means ± SE; young adults: n = 30 (15 females, 15 males); older adults: n = 25 (15 females, 10 males).

Figure S6. Figure S6A**.** Plasma total amino acids concentrations for young and older adults combined in the fasted (0-minute) and postprandial (300-minute) period after initiation of meal consumption for pork, eggs, black beans, and almonds. Different symbols indicate a significant difference between or among trials at each time point (Bonferroni adjusted, $P < 0.05$). * denotes a significant difference between pork or eggs vs. black beans. † denotes a significant difference between pork or eggs vs almonds. ‡ denotes a significant difference between pork vs eggs. # denotes a significant difference between pork vs. black beans. Figure S6B. Plasma total amino acids positive incremental area under the curve (iAUCpos) for pork (black bars), eggs (gray bars), black beans (white bars), and almonds (dashed black and white bars) for young and older adults combined. Different letters indicate a significant difference among trials (Bonferroni adjusted $P < 0.05$). All values are least squares means ± SE; n = 55 (30 (15 females, 15 males) young adults and 25 (15 females, 10 males) older adults).

Figure S7. Figure S7A**.** Plasma branched-chain amino acids concentrations for young and older adults combined in the fasted (0-minute) and postprandial (300-minute) period after initiation of meal consumption for pork, eggs, black beans, and almonds. Different symbols indicate a significant difference between or among trials at each time point (Bonferroni adjusted, $P < 0.05$). * denotes a significant difference between pork or eggs vs. black beans. † denotes a significant

difference between pork or eggs vs almonds. ‡ denotes a significant difference between pork vs eggs. Figure S7B. Plasma branched-chain amino acids positive incremental area under the curve (iAUCpos) for pork (black bars), eggs (gray bars), black beans (white bars), and almonds (dashed black and white bars) for young and older adults combined. Different letters indicate a significant difference among trials (Bonferroni adjusted $P < 0.05$). All values are least squares means ± SE; n = 55 (30 (15 females, 15 males) young adults and 25 (15 females, 10 males) older adults).

Figure S8. Figure 8A**.** Plasma leucine concentrations for young and older adults combined in the fasted (0-minute) and postprandial (300-minute) period after initiation of meal consumption for pork, eggs, black beans, and almonds. Different symbols indicate a significant difference between or among trials at each time point (Bonferroni adjusted, $P < 0.05$). * denotes a significant difference between pork or eggs vs. black beans. † denotes a significant difference between pork or eggs vs almonds. ‡ denotes a significant difference between pork vs eggs. Figure S8B. Plasma leucine positive incremental area under the curve (iAUCpos) for pork (black bars), eggs (gray bars), black beans (white bars), and almonds (dashed black and white bars) for young and older adults combined. Different letters indicate a significant difference among trials (Bonferroni adjusted $P < 0.05$). All values are least squares means ± SE; n = 55 (30 (15 females, 15 males) young adults and 25 (15 females, 10 males) older adults).

Effects of consuming ounce equivalent portions of animal- vs. plant-based Protein Foods, as defined by the Dietary Guidelines for Americans on essential amino acids bioavailability in young and older adults: crossover randomized controlled trials. Gavin Connolly

Figure S9. Figure S9A**.** Serum glucose concentrations for young and older adults combined in the fasted (0-minute) and postprandial (300-minute) period after initiation of meal consumption for pork, eggs, black beans, and almonds. Different symbols indicate a significant difference between or among trials at each time point (Bonferroni adjusted, $P < 0.05$). * denotes a significant difference between pork or eggs vs. black beans. Figure S9B. Serum glucose positive incremental area under the curve (iAUCpos) for pork (black bars), eggs (gray bars), black beans (white bars), and almonds (dashed black and white bars) for young and older adults combined. All values are least squares means ± SE; n = 55 (30 (15 females, 15 males) young adults and 25 (15 females, 10 males) older adults).

Figure S10. Figure S10A**.** Serum insulin concentrations for young and older adults combined in the fasted (0-minute) and postprandial (300-minute) period after initiation of meal consumption for pork, eggs, black beans, and almonds. Different symbols indicate a significant difference between or among trials at each time point (Bonferroni adjusted, $P < 0.05$). ‡ denotes a significant difference between pork vs eggs. $ denotes a significant difference between eggs vs. black beans. ¥ denotes a significant difference between pork vs. almonds. ¢ denotes a significant between black beans vs. almonds. Figure S10B. Serum insulin positive incremental area under the curve (iAUCpos) pork (black bars), eggs (gray bars), black beans (white bars), and almonds (dashed black and white bars) for young and older adults combined. All values are least squares means ± SE; n = 55 (30 (15 females, 15 males) young adults and 25 (15 females, 10 males) older adults).

Figure S11. Plasma total amino acids concentrations for females (Figure S11A) and males (Figure S11B) in the fasted (0-minute) and postprandial (300-minute) period after initiation of meal consumption for pork, eggs, black beans, and almonds. Different symbols indicate a significant difference between or among trials at each time point (Bonferroni adjusted, $P < 0.05$). * denotes a significant difference between pork or eggs vs. black beans. † denotes a significant difference between pork or eggs vs almonds. ‡ denotes a significant difference between pork vs eggs. ¥ denotes significant difference between pork vs. almonds. $ denotes a significant between eggs vs. black beans. # denotes a significant difference pork vs. black beans. Figure S11C. Plasma total amino acids positive incremental area under the curve (iAUCpos) pork (black bars), eggs (gray bars), black beans (white bars), and almonds (dashed black and white bars) for females and males. Different letters indicate a significant difference among trials within age group (Bonferroni adjusted $P < 0.05$). * denotes a significant difference between females and males for pork (Bonferroni adjusted $P < 0.05$). All values are least squares means ± SE; females: n = 30; males: n = 25.

Figure S12. Plasma branched-chain amino acids concentrations for females (Figure S12A) and males (Figure S12B) in the fasted (0-minute) and postprandial (300-minute) period after initiation of meal consumption for pork, eggs, black beans, and almonds. Different symbols indicate a significant difference between or among trials at each time point (Bonferroni adjusted, $P < 0.05$). * denotes a significant difference between pork or eggs vs. black beans. † denotes a

significant difference between pork or eggs vs almonds. ‡ denotes a significant difference between pork vs eggs. Figure S12C. Plasma branched-chain amino acids positive incremental area under the curve (iAUCpos) pork (black bars), eggs (gray bars), black beans (white bars), and almonds (dashed black and white bars) for females and males. Different letters indicate a significant difference among trials within age group (Bonferroni adjusted $P < 0.05$). ** denotes a significant difference between females and males for pork (Bonferroni adjusted $P < 0.01$). All values are least squares means ± SE; females: n = 30; males: n = 25.

Figure S13. Plasma leucine concentrations for females (Figure S13A) and males (Figure S13B) in the fasted (0-minute) and postprandial (300-minute) period after initiation of meal consumption for pork, eggs, black beans, and almonds. Different symbols indicate a significant difference between or among trials at each time point (Bonferroni adjusted, $P < 0.05$). * denotes a significant difference between pork or eggs vs. black beans. † denotes a significant difference between pork or eggs vs almonds. ‡ denotes a significant difference between pork vs eggs. ¥ denotes significant difference between pork vs. almonds. # denotes a significant difference between pork vs. black beans. Figure S13C. Plasma leucine positive incremental area under the curve (iAUCpos) for pork (black bars), eggs (gray bars), black beans (white bars), and almonds (dashed black and white bars) for females and males. Different letters indicate a significant difference among trials within age group (Bonferroni adjusted $P < 0.05$). ** denotes a significant difference between females and males for pork (Bonferroni adjusted $P < 0.01$). All values are least squares means ± SE; females: n = 30; males: n = 25.

Effects of consuming ounce equivalent portions of animal- vs. plant-based Protein Foods, as defined by the Dietary Guidelines for Americans on essential amino acids bioavailability in young and older adults: crossover randomized controlled trials. Gavin Connolly

Figure S14. Serum glucose concentrations for females (Figure S14A) and males (Figure S14B) in the fasted (0-minute) and postprandial (300-minute) period after initiation of meal consumption for pork, eggs, black beans, and almonds. Different symbols indicate a significant difference between or among trials at each time point (Bonferroni adjusted, $P < 0.05$). $ denotes a significant difference between eggs vs. black beans. # denotes a significant difference between pork vs. black beans. Figure S14C. Serum glucose positive incremental area under the curve (iAUCpos) for pork (black bars), eggs (gray bars), black beans (white bars), and almonds (dashed black and white bars) for females and males. All values are least squares means ± SE; females: n = 30; males: n = 25.

Figure S15. Serum insulin concentrations for females (Figure S15A) and males (Figure S15B) in the fasted (0-minute) and postprandial (300-minute) period after initiation of meal consumption for pork, eggs, black beans, and almonds. Different symbols indicate a significant difference between or among trials at each time point (Bonferroni adjusted, $P < 0.05$). $ denotes a significant difference between eggs vs. black beans. ¥ denotes a significant difference between pork vs. almonds. ¢ denotes a significant between black beans vs. almonds. Figure S15C. Serum insulin positive incremental area under the curve (iAUCpos) for pork (black bars), eggs (gray bars), black beans (white bars), and almonds (dashed black and white bars) for females and males. All values are least squares means ± SE; females: n = 30; males: n = 25.

Effects of consuming ounce equivalent portions of animal- vs. plant-based Protein Foods, as defined by the Dietary Guidelines for Americans on essential amino acids bioavailability in young and older adults: crossover randomized controlled trials. Gavin Connolly

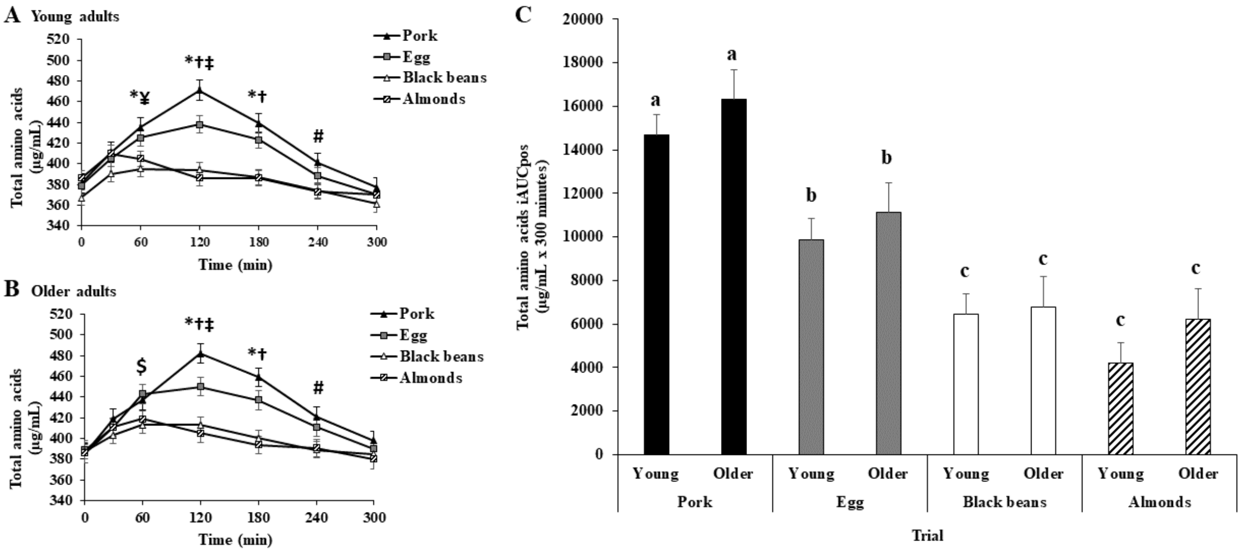

Figure S1. Plasma total amino acids concentrations for young adults (Figure S1A) and older adults (Figure S1B) in the fasted (0-minute) and postprandial (300-minute) period after initiation of meal consumption for pork, eggs, black beans, and almonds. Different symbols indicate a significant difference between or among trials at each time point (Bonferroni adjusted, $P < 0.05$). * denotes a significant difference between pork or eggs vs. black beans. † denotes a significant difference between pork or eggs vs almonds. ‡ denotes a significant difference between pork vs eggs. ¥ denotes significant difference between pork vs. almonds. # denotes a significant difference between pork vs. black beans. Figure S1C. Plasma total amino acids positive incremental area under the curve (iAUCpos) for pork (black bars), eggs (gray bars), black beans (white bars), and almonds (dashed black and white bars) for young and older adults. Different letters indicate a significant difference among trials within age group (Bonferroni adjusted $P < 0.05$). All values are least squares means ± SE; young adults: n = 30 (15 females, 15 males); older adults: n = 25 (15 females, 10 males).

Effects of consuming ounce equivalent portions of animal- vs. plant-based Protein Foods, as defined by the Dietary Guidelines for Americans on essential amino acids bioavailability in young and older adults: crossover randomized controlled trials. Gavin Connolly

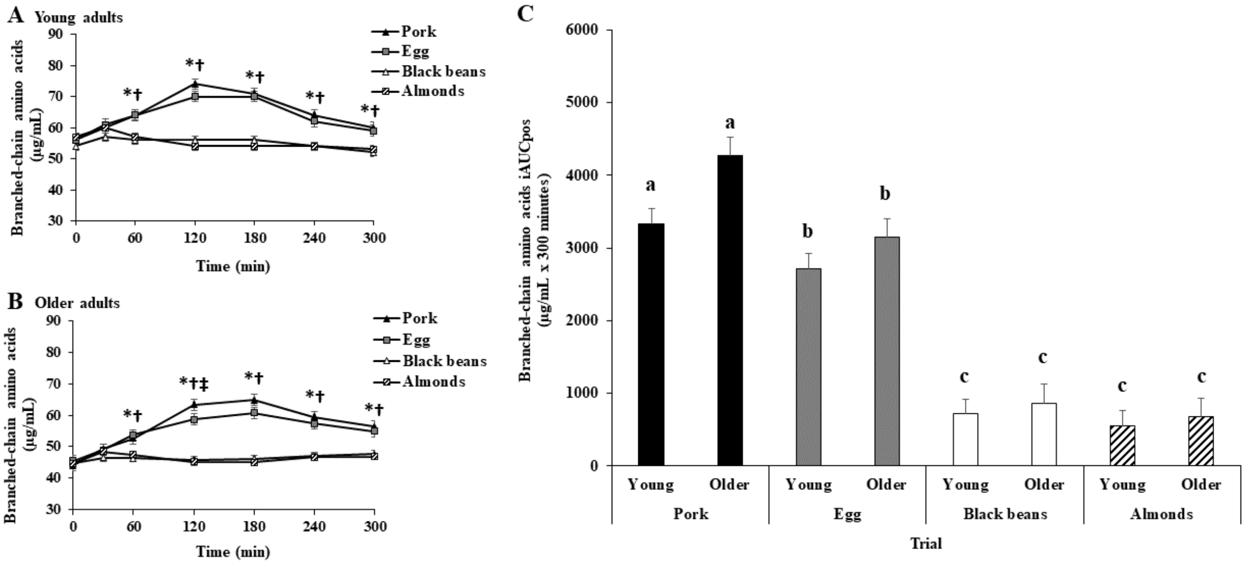

Figure S2. Plasma branched-chain amino acids concentrations for young adults (Figure S2A) and older adults (Figure S2B) in the fasted (0-minute) and postprandial (300-minute) period after initiation of meal consumption for pork, eggs, black beans, and almonds. Different symbols indicate a significant difference between or among trials at each time point (Bonferroni adjusted, $P < 0.05$). * denotes a significant difference between pork or eggs vs. black beans. † denotes a significant difference between pork or eggs vs almonds. ‡ denotes a significant difference between pork vs eggs. ¥ denotes significant difference between pork vs. almonds. Figure S2C. Plasma branched-chain amino acids positive incremental area under the curve (iAUCpos) for pork (black bars), eggs (gray bars), black beans (white bars), and almonds (dashed black and white bars) for young and older adults. Different letters indicate a significant difference among trials within age group (Bonferroni adjusted $P < 0.05$). All values are least squares means ± SE; young adults: n = 30 (15 females, 15 males); older adults: n = 25 (15 females, 10 males).

Effects of consuming ounce equivalent portions of animal- vs. plant-based Protein Foods, as defined by the Dietary Guidelines for Americans on essential amino acids bioavailability in young and older adults: crossover randomized controlled trials. Gavin Connolly

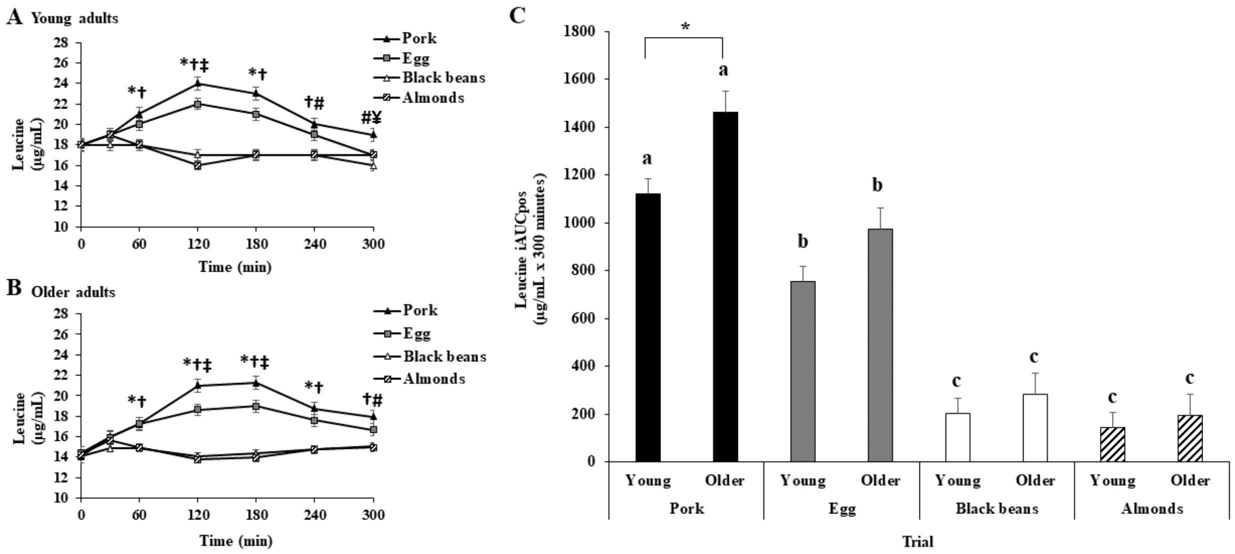

Figure S3. Plasma leucine concentrations for young adults (Figure S3A) and older adults (Figure S3B) in the fasted (0-minute) and postprandial (300-minute) period after initiation of meal consumption for pork, eggs, black beans, and almonds. Different symbols indicate a significant difference between or among trials at each time point (Bonferroni adjusted, $P < 0.05$). * denotes a significant difference between pork or eggs vs. black beans. † denotes a significant difference between pork or eggs vs almonds. ‡ denotes a significant difference between pork vs eggs. ¥ denotes significant difference between pork vs. almonds. # denotes a significant difference between pork vs. black beans. Figure S3C. Plasma leucine positive incremental area under the curve (iAUCpos) for pork (black bars), eggs (gray bars), black beans (white bars), and almonds (dashed black and white bars) for young and older adults. Different letters indicate a significant difference among trials within age group (Bonferroni adjusted $P < 0.05$). * denotes a significant difference between young and older adults for pork (Bonferroni adjusted $P < 0.05$). All values

Effects of consuming ounce equivalent portions of animal- vs. plant-based Protein Foods, as defined by the Dietary Guidelines for Americans on essential amino acids bioavailability in young and older adults: crossover randomized controlled trials. Gavin Connolly

are least squares means ± SE; young adults: n = 30 (15 females, 15 males); older adults: n = 25 (15 females, 10 males).

Effects of consuming ounce equivalent portions of animal- vs. plant-based Protein Foods, as defined by the Dietary Guidelines for Americans on essential amino acids bioavailability in young and older adults: crossover randomized controlled trials. Gavin Connolly

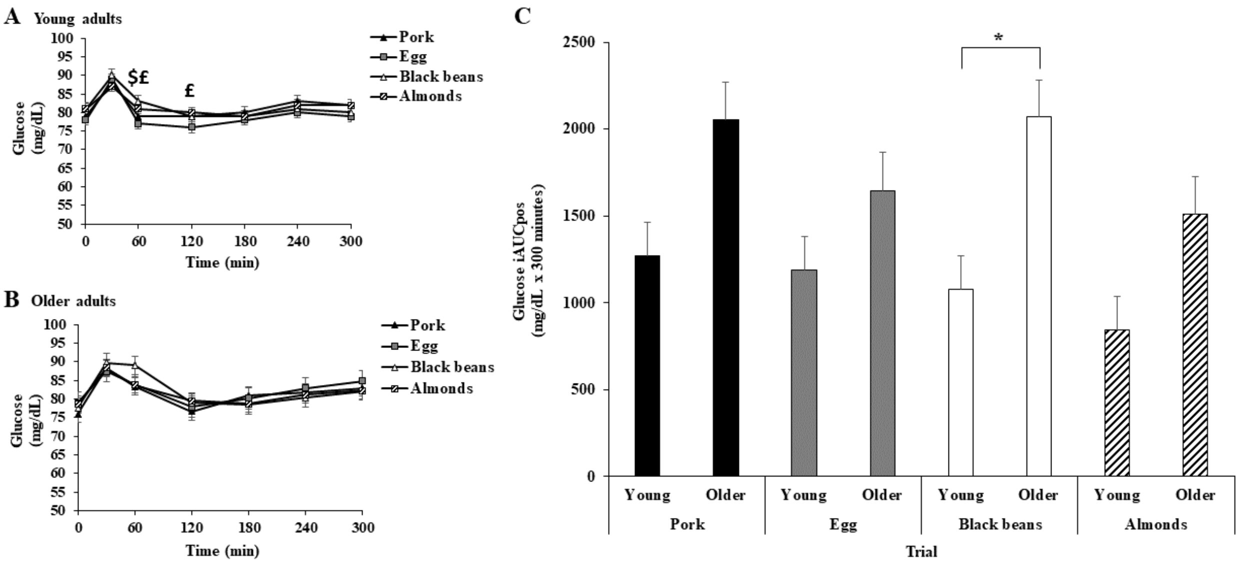

Figure S4. Serum glucose concentrations for young adults (Figure S4A) and older adults (Figure S4B) in the fasted (0-minute) and postprandial (300-minute) period after initiation of meal consumption for pork, eggs, black beans, and almonds. Different symbols indicate a significant difference between or among trials at each time point (Bonferroni adjusted, $P < 0.05$). $ denotes a significant difference between eggs vs. black beans. £ denotes a significant difference between eggs vs. almonds. Figure S4C. Serum glucose positive incremental area under the curve (iAUCpos) for pork (black bars), eggs (gray bars), black beans (white bars), and almonds (dashed black and white bars) for young and older adults. * denotes a significant difference between young and older adults for black beans (Bonferroni adjusted $P < 0.05$). All values are least squares means ± SE; young adults: n = 30 (15 females, 15 males); older adults: n = 25 (15 females, 10 males).

Effects of consuming ounce equivalent portions of animal- vs. plant-based Protein Foods, as defined by the Dietary Guidelines for Americans on essential amino acids bioavailability in young and older adults: crossover randomized controlled trials. Gavin Connolly

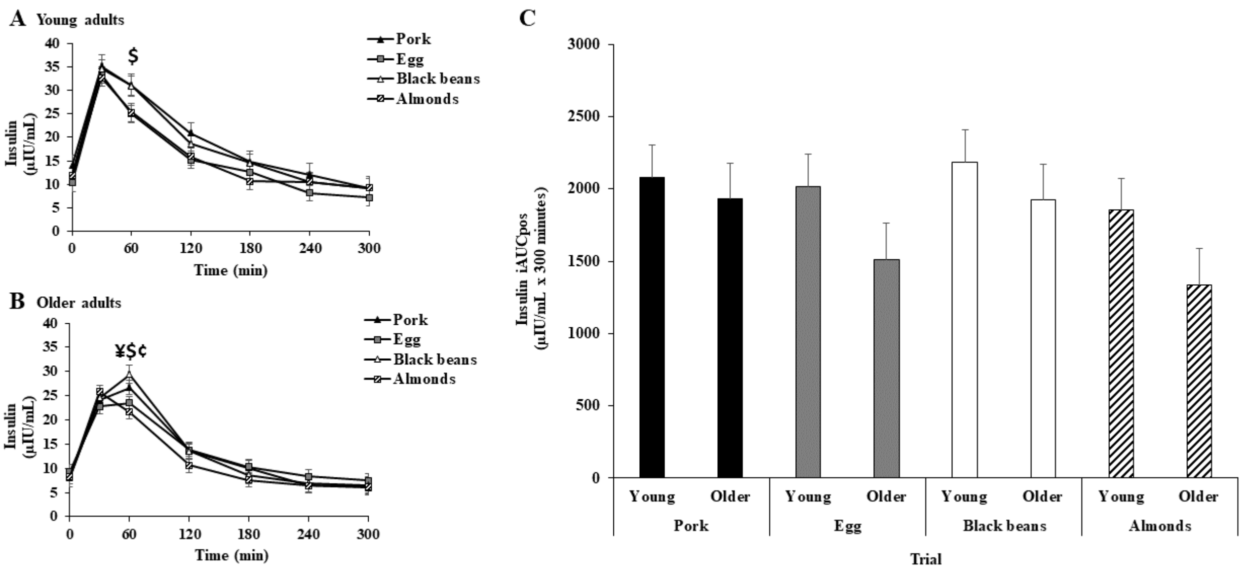

Figure S5. Serum insulin concentrations for young adults (Figure S5A) and older adults (Figure S5B) in the fasted (0-minute) and postprandial (300-minute) period after initiation of meal consumption for pork, eggs, black beans, and almonds. Different symbols indicate a significant difference between or among trials at each time point (Bonferroni adjusted, $P < 0.05$). $ denotes a significant difference between eggs vs. black beans. ¥ denotes a significant difference between pork vs. almonds. ¢ denotes a significant between black beans vs. almonds. Figure S5C. Serum insulin positive incremental area under the curve (iAUCpos) for pork (black bars), eggs (gray bars), black beans (white bars), and almonds (dashed black and white bars) for young and older adults. All values are least squares means ± SE; young adults: n = 30 (15 females, 15 males); older adults: n = 25 (15 females, 10 males).

Effects of consuming ounce equivalent portions of animal- vs. plant-based Protein Foods, as defined by the Dietary Guidelines for Americans on essential amino acids bioavailability in young and older adults: crossover randomized controlled trials. Gavin Connolly

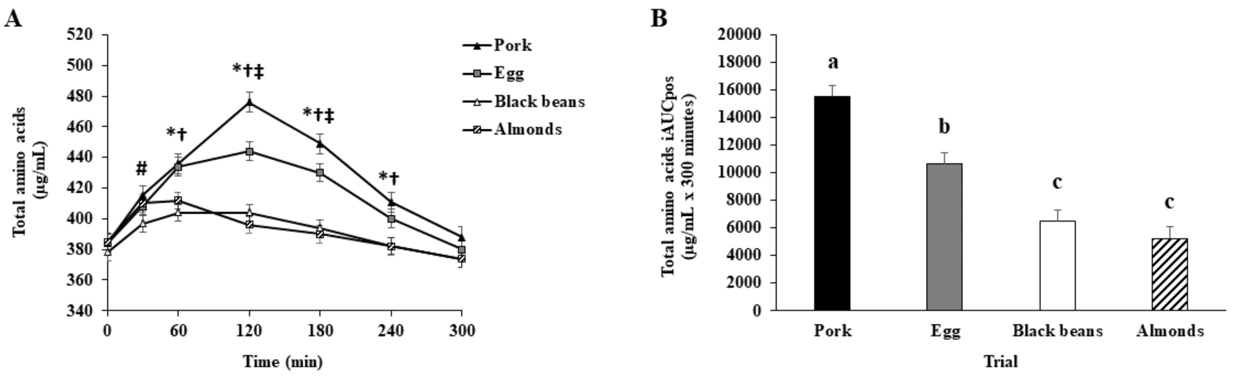

Figure S6. Figure S6A**.** Plasma total amino acids concentrations for young and older adults combined in the fasted (0-minute) and postprandial (300-minute) period after initiation of meal consumption for pork, eggs, black beans, and almonds. Different symbols indicate a significant difference between or among trials at each time point (Bonferroni adjusted, $P < 0.05$). * denotes a significant difference between pork or eggs vs. black beans. † denotes a significant difference between pork or eggs vs almonds. ‡ denotes a significant difference between pork vs eggs. # denotes a significant difference between pork vs. black beans. Figure S6B. Plasma total amino acids positive incremental area under the curve (iAUCpos) for pork (black bars), eggs (gray bars), black beans (white bars), and almonds (dashed black and white bars) for young and older adults combined. Different letters indicate a significant difference among trials (Bonferroni adjusted $P < 0.05$). All values are least squares means ± SE; n = 55 (30 (15 females, 15 males) young adults and 25 (15 females, 10 males) older adults).

Effects of consuming ounce equivalent portions of animal- vs. plant-based Protein Foods, as defined by the Dietary Guidelines for Americans on essential amino acids bioavailability in young and older adults: crossover randomized controlled trials. Gavin Connolly

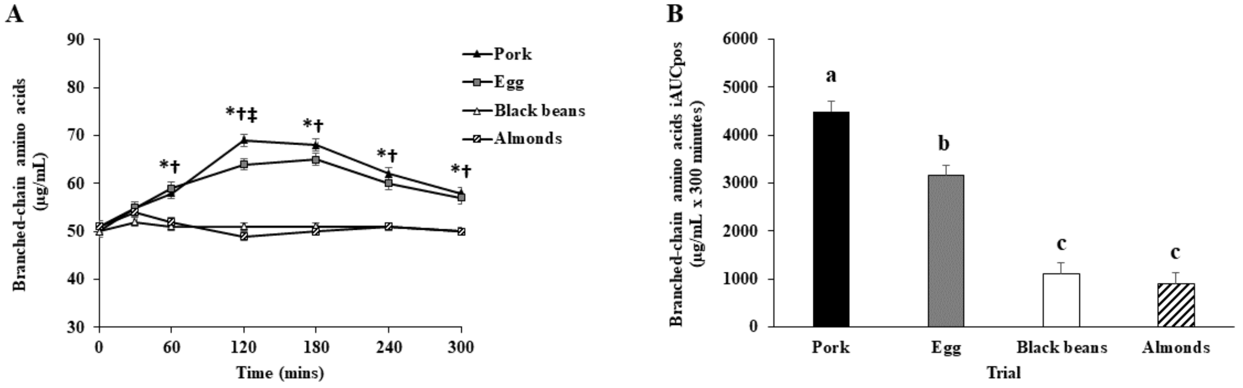

Figure S7. Figure S7A. Plasma branched-chain amino acids concentrations for young and older adults combined in the fasted (0-minute) and postprandial (300-minute) period after initiation of meal consumption for pork, eggs, black beans, and almonds. Different symbols indicate a significant difference between or among trials at each time point (Bonferroni adjusted, $P < 0.05$). * denotes a significant difference between pork or eggs vs. black beans. † denotes a significant difference between pork or eggs vs almonds. ‡ denotes a significant difference between pork vs eggs. Figure S7B. Plasma branched-chain amino acids positive incremental area under the curve (iAUCpos) for pork (black bars), eggs (gray bars), black beans (white bars), and almonds (dashed black and white bars) for young and older adults combined. Different letters indicate a significant difference among trials (Bonferroni adjusted $P < 0.05$). All values are least squares means ± SE; n = 55 (30 (15 females, 15 males) young adults and 25 (15 females, 10 males) older adults).

Effects of consuming ounce equivalent portions of animal- vs. plant-based Protein Foods, as defined by the Dietary Guidelines for Americans on essential amino acids bioavailability in young and older adults: crossover randomized controlled trials. Gavin Connolly

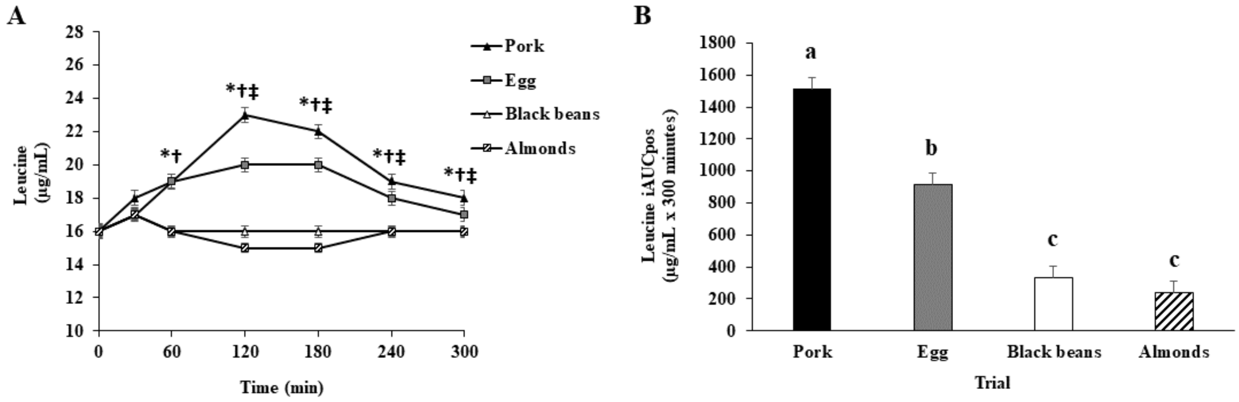

Figure S8. Figure S8A**.** Plasma leucine concentrations for young and older adults combined in the fasted (0-minute) and postprandial (300-minute) period after initiation of meal consumption for pork, eggs, black beans, and almonds. Different symbols indicate a significant difference between or among trials at each time point (Bonferroni adjusted, $P < 0.05$). * denotes a significant difference between pork or eggs vs. black beans. † denotes a significant difference between pork or eggs vs almonds. ‡ denotes a significant difference between pork vs eggs. Figure S8B. Plasma leucine positive incremental area under the curve (iAUCpos) for pork (black bars), eggs (gray bars), black beans (white bars), and almonds (dashed black and white bars) for young and older adults combined. Different letters indicate a significant difference among trials (Bonferroni adjusted $P < 0.05$). All values are least squares means ± SE; n = 55 (30 (15 females, 15 males) young adults and 25 (15 females, 10 males) older adults).

Effects of consuming ounce equivalent portions of animal- vs. plant-based Protein Foods, as defined by the Dietary Guidelines for Americans on essential amino acids bioavailability in young and older adults: crossover randomized controlled trials. Gavin Connolly

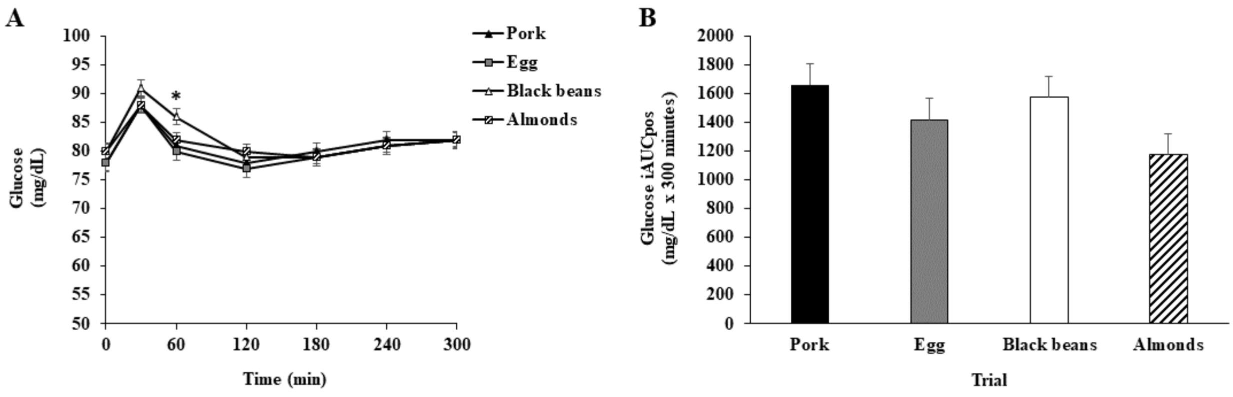

Figure S9. Figure S9A**.** Serum glucose concentrations for young and older adults combined in the fasted (0-minute) and postprandial (300-minute) period after initiation of meal consumption for pork, eggs, black beans, and almonds. Different symbols indicate a significant difference between or among trials at each time point (Bonferroni adjusted, $P < 0.05$). * denotes a significant difference between pork or eggs vs. black beans. Figure S9B. Serum glucose positive incremental area under the curve (iAUCpos) for pork (black bars), eggs (gray bars), black beans (white bars), and almonds (dashed black and white bars) for young and older adults combined. All values are least squares means ± SE; n = 55 (30 (15 females, 15 males) young adults and 25 (15 females, 10 males) older adults).

Effects of consuming ounce equivalent portions of animal- vs. plant-based Protein Foods, as defined by the Dietary Guidelines for Americans on essential amino acids bioavailability in young and older adults: crossover randomized controlled trials. Gavin Connolly

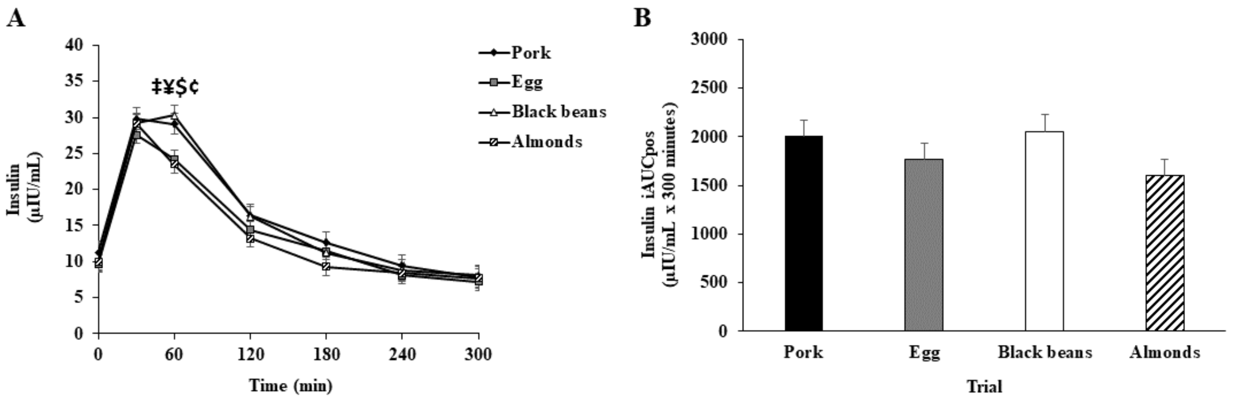

Figure S10. Figure 10A**.** Serum insulin concentrations for young and older adults combined in the fasted (0-minute) and postprandial (300-minute) period after initiation of meal consumption for pork, eggs, black beans, and almonds. Different symbols indicate a significant difference between or among trials at each time point (Bonferroni adjusted, $P < 0.05$). ‡ denotes a significant difference between pork vs eggs. $ denotes a significant difference between eggs vs. black beans. ¥ denotes a significant difference between pork vs. almonds. ¢ denotes a significant between black beans vs. almonds. Figure S10B. Serum insulin positive incremental area under the curve (iAUCpos) for pork (black bars), eggs (gray bars), black beans (white bars), and almonds (dashed black and white bars) for young and older adults combined. All values are least squares means ± SE; n = 55 (30 (15 females, 15 males) young adults and 25 (15 females, 10 males) older adults).

Effects of consuming ounce equivalent portions of animal- vs. plant-based Protein Foods, as defined by the Dietary Guidelines for Americans on essential amino acids bioavailability in young and older adults: crossover randomized controlled trials. Gavin Connolly

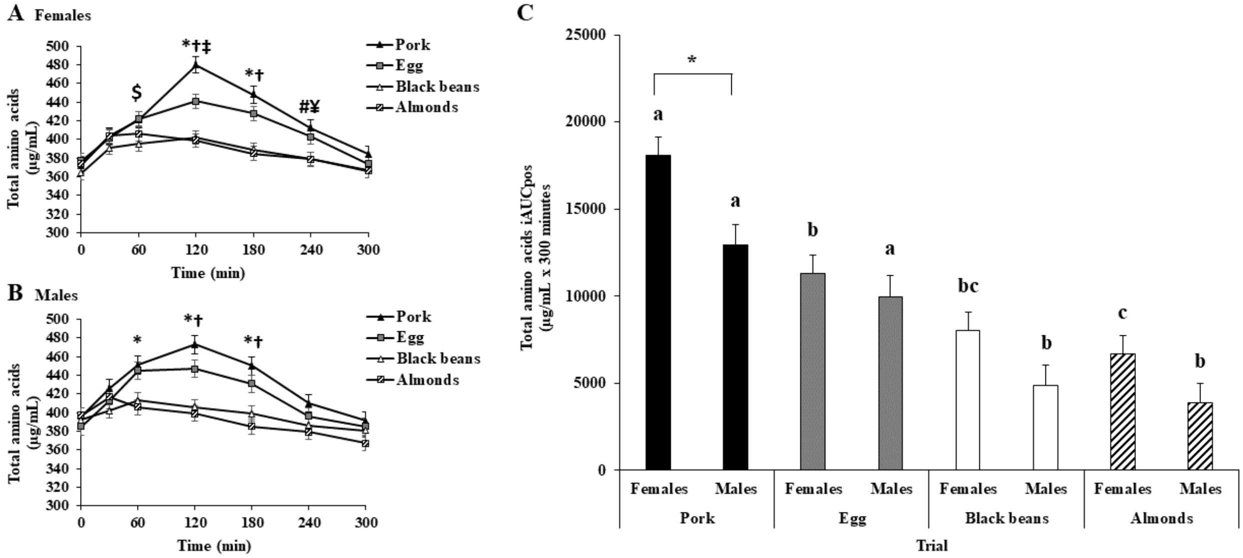

Figure S11. Plasma total amino acids concentrations for females (Figure S11A) and males (Figure S11B) in the fasted (0-minute) and postprandial (300-minute) period after initiation of meal consumption for pork, eggs, black beans, and almonds. Different symbols indicate a significant difference between or among trials at each time point (Bonferroni adjusted, *P* < 0.05). * denotes a significant difference between pork or eggs vs. black beans. † denotes a significant difference between pork or eggs vs almonds. ‡ denotes a significant difference between pork vs eggs. ¥ denotes significant difference between pork vs. almonds. $ denotes a significant between eggs vs. black beans. # denotes a significant difference pork vs. black beans. Figure S11C. Plasma total amino acids positive incremental area under the curve (iAUCpos) for pork (black bars), eggs (gray bars), black beans (white bars), and almonds (dashed black and white bars) for females and males. Different letters indicate a significant difference among trials within age group (Bonferroni adjusted *P* < 0.05). * denotes a significant difference between females and males for pork (Bonferroni adjusted *P* < 0.05). All values are least squares means ± SE; females: n = 30; males: n = 25.

Effects of consuming ounce equivalent portions of animal- vs. plant-based Protein Foods, as defined by the Dietary Guidelines for Americans on essential amino acids bioavailability in young and older adults: crossover randomized controlled trials. Gavin Connolly

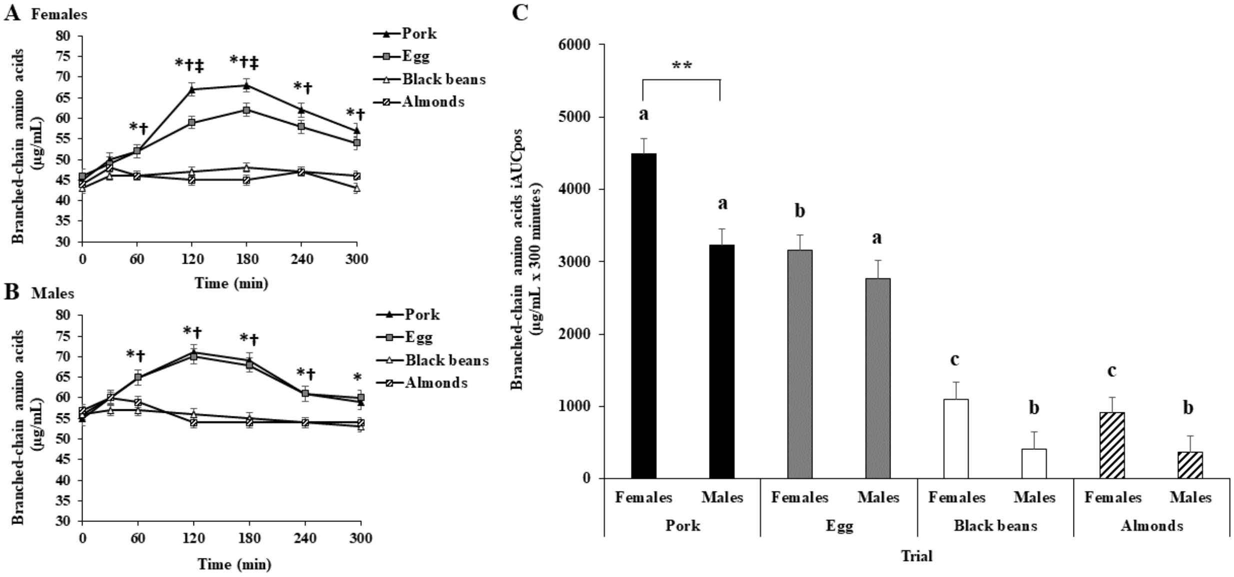

Figure S12. Plasma branched-chain amino acids concentrations for females (Figure S12A) and males (Figure S12B) in the fasted (0-minute) and postprandial (300-minute) period after initiation of meal consumption for pork, eggs, black beans, and almonds. Different symbols indicate a significant difference between or among trials at each time point (Bonferroni adjusted, $P < 0.05$). * denotes a significant difference between pork or eggs vs. black beans. † denotes a significant difference between pork or eggs vs almonds. ‡ denotes a significant difference between pork vs eggs. Figure S12C. Plasma branched-chain amino acids positive incremental area under the curve (iAUCpos) for pork (black bars), eggs (gray bars), black beans (white bars), and almonds (dashed black and white bars) for females and males. Different letters indicate a significant difference among trials within age group (Bonferroni adjusted $P < 0.05$). ** denotes a significant difference between females and males for pork (Bonferroni adjusted $P < 0.01$). All values are least squares means ± SE; females: n = 30; males: n = 25.

Effects of consuming ounce equivalent portions of animal- vs. plant-based Protein Foods, as defined by the Dietary Guidelines for Americans on essential amino acids bioavailability in young and older adults: crossover randomized controlled trials. Gavin Connolly

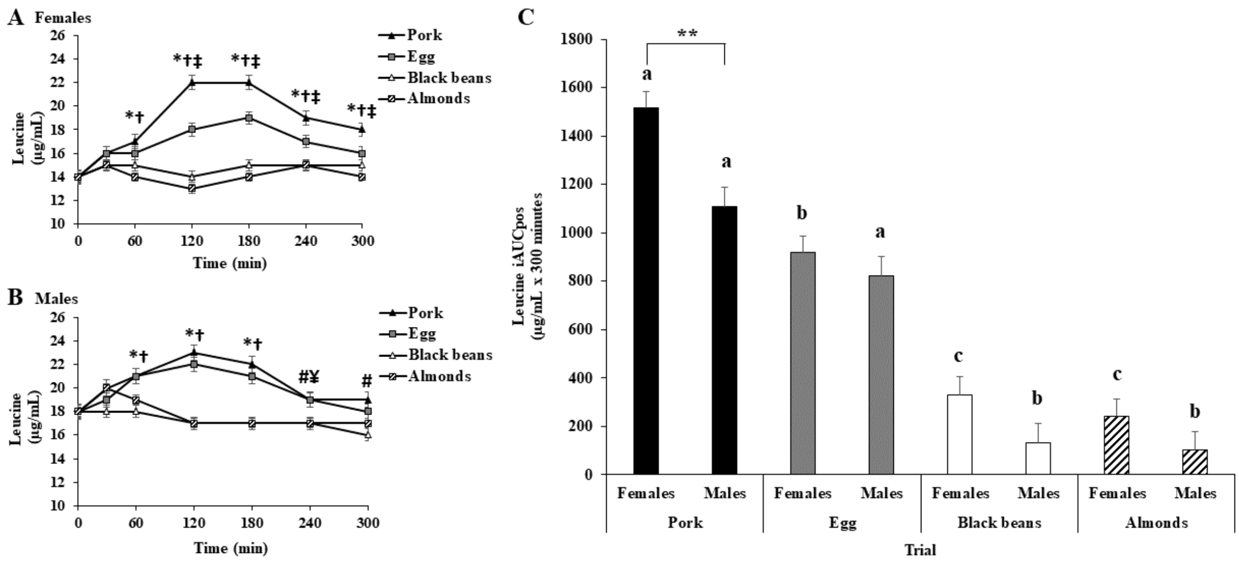

Figure S13. Plasma leucine concentrations for females (Figure S13A) and males (Figure S13B) in the fasted (0-minute) and postprandial (300-minute) period after initiation of meal consumption for pork, eggs, black beans, and almonds. Different symbols indicate a significant difference between or among trials at each time point (Bonferroni adjusted, $P < 0.05$). * denotes a significant difference between pork or eggs vs. black beans. † denotes a significant difference between pork or eggs vs almonds. ‡ denotes a significant difference between pork vs eggs. ¥ denotes significant difference between pork vs. almonds. # denotes a significant difference between pork vs. black beans. Figure S13C. Plasma leucine positive incremental area under the curve (iAUCpos) for pork (black bars), eggs (gray bars), black beans (white bars), and almonds (dashed black and white bars) for females and males. Different letters indicate a significant difference among trials within age group (Bonferroni adjusted $P < 0.05$). ** denotes a significant difference between females and males for pork (Bonferroni adjusted $P < 0.01$). All values are least squares means ± SE; females: n = 30; males: n = 25.

Effects of consuming ounce equivalent portions of animal- vs. plant-based Protein Foods, as defined by the Dietary Guidelines for Americans on essential amino acids bioavailability in young and older adults: crossover randomized controlled trials. Gavin Connolly

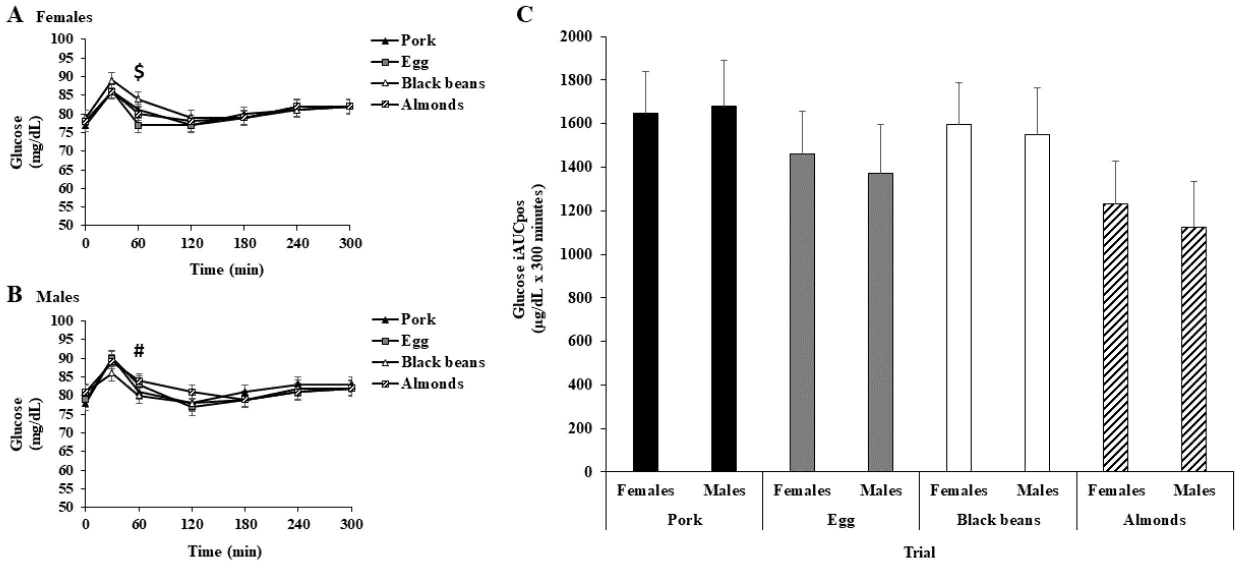

Figure S14. Serum glucose concentrations for females (Figure S14A) and males (Figure S14B) in the fasted (0-minute) and postprandial (300-minute) period after initiation of meal consumption for pork, eggs, black beans, and almonds. Different symbols indicate a significant difference between or among trials at each time point (Bonferroni adjusted, $P < 0.05$). $ denotes a significant difference between eggs vs. black beans. # denotes a significant difference between pork vs. black beans. Figure S14C. Serum glucose positive incremental area under the curve (iAUCpos) for pork (black bars), eggs (gray bars), black beans (white bars), and almonds (dashed black and white bars) for females and males. All values are least squares means ± SE; females: n = 30; males: n = 25.

Effects of consuming ounce equivalent portions of animal- vs. plant-based Protein Foods, as defined by the Dietary Guidelines for Americans on essential amino acids bioavailability in young and older adults: crossover randomized controlled trials. Gavin Connolly

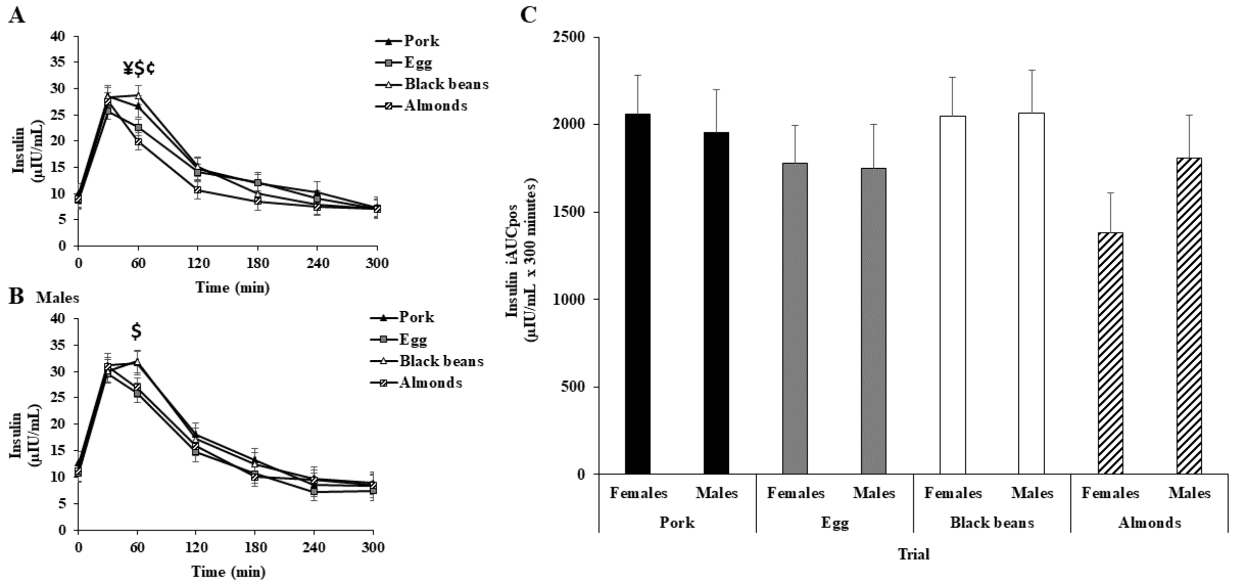

Figure S15. Serum insulin concentrations for females (Figure S15A) and males (Figure S15B) in the fasted (0-minute) and postprandial (300-minute) period after initiation of meal consumption for pork, eggs, black beans, and almonds. Different symbols indicate a significant difference between or among trials at each time point (Bonferroni adjusted, $P < 0.05$). $ denotes a significant difference between eggs vs. black beans. ¥ denotes a significant difference between pork vs. almonds. ¢ denotes a significant between black beans vs. almonds. Figure S15C. Serum insulin positive incremental area under the curve (iAUCpos) for pork (black bars), eggs (gray bars), black beans (white bars), and almonds (dashed black and white bars) for females and males. All values are least squares means ± SE; females: n = 30; males: n = 25.
