# Supplementary material for: Effects of Consuming Ounce-Equivalent Portions of Animal- vs. Plant-Based Protein Foods, as Defined by the Dietary Guidelines for Americans on Essential Amino Acids Bioavailability in Young and Older Adults: Two Cross-Over Randomized Controlled Trials
Source: Nutrients. 2023 Jun 25;15(13):2870. doi: 10.3390/nu15132870 (PMC10343739; doi:10.3390/nu15132870)
Supplement: Supplementary file 1 [file nutrients-15-02870-s001.zip › Supplementary Tables.pdf]

Effects of consuming ounce equivalent portions of animal- vs. plant-based Protein Foods, as defined by the Dietary Guidelines for Americans on essential amino acids bioavailability in young and older adults: crossover randomized controlled trials. Gavin Connolly

Title: Effects of consuming ounce equivalent portions of animal- vs. plant-based Protein Foods, as defined by the Dietary Guidelines for Americans on essential amino acids bioavailability in young and older adults: crossover randomized controlled trials

First Author: Gavin Connolly

Online Supplementary Tables

Table S1. Energy and macronutrients contents of the food items in the controlled dinner.

Table S2. Weight and energy and macronutrients contents of the food items in the test salad.

Table S3. Fasting and postprandial amino acids, glucose, and insulin concentrations and positive incremental areas under the curve for young adults.

Table S4. Fasting and postprandial amino acids, glucose, and insulin concentrations and positive incremental areas under the curve for older adults.

Table S5. Fasting and postprandial amino acids, glucose, and insulin concentrations and positive incremental areas under the curve for young and older adults combined.

Table S6. Fasting and postprandial amino acids, glucose, and insulin concentrations and positive incremental areas under the curve for females.

Table S7. Fasting and postprandial amino acids, glucose, and insulin concentrations and positive incremental areas under the curve for males.

Effects of consuming ounce equivalent portions of animal- vs. plant-based Protein Foods, as defined by the Dietary Guidelines for Americans on essential amino acids bioavailability in young and older adults: crossover randomized controlled trials. Gavin Connolly

Table S1. Energy and macronutrients contents of the food items in the controlled dinner.

**Food item**	**Energy (kcal)**	**Fat (g)**	**CHO (g)**	**Protein (g)**
Lean Cuisine Favorites Chicken Fettucine (two packs)	580	11.6	85.2	31.9
Hawaiian roll	100	2.5	16.9	2.6
Butter, regular, salted (two packets)	68	7.7	0.0	0.1
Medium apple, fresh, with skin	95	0.3	25.1	0.5
¾ cup of yellow corn	96	0.8	22.9	3.0
Milk, 1% fat, 270 mL	113	2.6	13.5	9.1
Total	1052	25.5	163.6	47.2

Effects of consuming ounce equivalent portions of animal- vs. plant-based Protein Foods, as defined by the Dietary Guidelines for Americans on essential amino acids bioavailability in young and older adults: crossover randomized controlled trials. Gavin Connolly

Table S2. Weight and energy and macronutrients contents of the food items in the test salad.

**Food item**	**Weight (g)**	**Energy (kcal)**	**Fat (g)**	**CHO (g)**	**Protein (g)**
Romaine lettuce	20	3	0.1	0.7	0.2
Mushrooms, raw	25	6	0.1	0.8	0.8
Croutons	12	49	0.8	8.8	1.4
Baby spinach, raw	50	12	0.2	1.8	1.4
Carrots, raw	62	25	0.1	5.9	0.6
Tomato, raw	70	13	0.1	2.7	0.6
Broccoli, raw	34	12	0.1	2.3	1.0
Balsamic vinaigrette dressing	31	99	10	2.8	0
Total		218	11.5	25.8	6

Effects of consuming ounce equivalent portions of animal- vs. plant-based Protein Foods, as defined by the Dietary Guidelines for Americans on essential amino acids bioavailability in young and older adults: crossover randomized controlled trials. Gavin Connolly

Table S3. Fasting and postprandial amino acids, glucose, and insulin concentrations and positive incremental areas under the curve for young adults.

	**Time (mins)**							**iAUCpos**
	**0**	**30**	**60**	**120**	**180**	**240**	**300**	
				µg/mL[1]				µg/mL*300 min[2]
**TAAs**								
**Pork**	396 ± 10^{a}	426 ± 10^{a}	451 ± 10^{a}	473 ± 10^{a}	450 ± 10^{a}	410 ± 10^{a}	391 ± 10^{a}	14671 ± 939^{a}
**Egg**	385 ± 9^{a}	413 ± 9^{a}	445 ± 9^{ab}	447 ± 9^{b}	431 ± 9^{a}	396 ± 9^{ab}	385 ± 9^{a}	9870 ± 963^{b}
**Black beans**	392 ± 8^{a}	402 ± 8^{a}	413 ± 8^{c}	406 ± 8^{c}	399 ± 8^{b}	386 ± 8^{b}	381 ± 8^{a}	6447 ± 941^{c}
**Almonds**	397 ± 8^{a}	417 ± 8^{a}	406 ± 8^{bc}	399 ± 8^{c}	385 ± 8^{b}	379 ± 8^{ab}	367 ± 8^{a}	4189 ± 940^{c}
**EAAs**								
**Pork**	129 ± 3^{a}	141 ± 3^{a}	152 ± 3^{a}	171 ± 3^{a}	161 ± 3^{a}	144 ± 3^{a}	134 ± 3^{a}	7346 ± 401^{a}
**Egg**	130 ± 3^{a}	139 ± 3^{a}	147 ± 3^{a}	158 ± 3^{b}	153 ± 3^{a}	137 ± 3^{a}	130 ± 3^{a}	4689 ± 413^{b}
**Black beans**	124 ± 2^{a}	131 ± 2^{b}	132 ± 2^{b}	132 ± 2^{c}	131 ± 2^{b}	125 ± 2^{b}	121 ± 2^{b}	2023 ± 402^{c}
**Almonds**	130 ± 2^{a}	137 ± 2^{ab}	133 ± 2^{b}	126 ± 2^{c}	127 ± 2^{b}	124 ± 2^{b}	122 ± 2^{b}	1184 ± 401^{c}
**BCAAs**								
**Pork**	56 ± 2^{a}	60 ± 2^{a}	64 ± 2^{a}	74 ± 2^{a}	71 ± 2^{a}	64 ± 2^{a}	60 ± 2^{a}	3336 ± 200^{a}
**Egg**	56 ± 2^{a}	61 ± 2^{a}	64 ± 2^{a}	70 ± 2^{a}	70 ± 2^{a}	62 ± 2^{a}	59 ± 2^{a}	2719 ± 206^{b}
**Black beans**	54 ± 1^{a}	57 ± 1^{a}	56 ± 1^{b}	56 ± 1^{b}	56 ± 1^{b}	54 ± 1^{b}	52 ± 1^{b}	716 ± 201^{c}
**Almonds**	57 ± 1^{a}	60 ± 1^{a}	57 ± 1^{b}	54 ± 1^{b}	54 ± 1^{b}	54 ± 1^{b}	53 ± 1^{b}	558 ± 200^{c}
**Leucine**								
**Pork**	18 ± 1^{a}	19 ± 1^{a}	21 ± 1^{a}	24 ± 1^{a}	23 ± 1^{a}	20 ± 1^{a}	19 ± 1^{a}	1118 ± 63^{a}
**Egg**	18 ± 1^{a}	19 ± 1^{a}	20 ± 1^{a}	22 ± 1^{b}	21 ± 1^{a}	19 ± 1^{ab}	17 ± 1^{ab}	753 ± 65^{b}
**Black beans**	18 ± 1^{a}	18 ± 1^{a}	18 ± 1^{b}	17 ± 1^{c}	17 ± 1^{b}	17 ± 1^{bc}	16 ± 1^{b}	201 ± 64^{c}
**Almonds**	18 ± 1^{a}	19 ± 1^{a}	18 ± 1^{b}	16 ± 1^{c}	17 ± 1^{b}	17 ± 1^{c}	17 ± 1^{b}	143 ± 63^{c}
				mg/dL				mg/dL*300 min
**Glucose**								
**Pork**	79 ± 2^{a}	88 ± 2^{a}	79 ± 2^{abc}	79 ± 2^{abc}	80 ± 2^{a}	83 ± 2^{a}	82 ± 2^{a}	1262 ± 164^{a}
**Egg**	78 ± 1^{a}	89 ± 1^{a}	77 ± 1^{ab}	76 ± 1^{ab}	78 ± 1^{a}	80 ± 1^{a}	79 ± 1^{a}	1165 ± 169^{a}
**Black beans**	81 ± 2^{a}	90 ± 2^{a}	83 ± 2^{ac}	79 ± 2^{abc}	79 ± 2^{a}	81 ± 2^{a}	80 ± 2^{a}	1044 ± 165^{a}
**Almonds**	81 ± 1^{a}	87 ± 1^{a}	81 ± 1^{abc}	80 ± 1^{ac}	79 ± 1^{a}	82 ± 1^{a}	82 ± 1^{a}	889 ± 164^{a}
				µIU/mL				µIU/mL*300 min

Effects of consuming ounce equivalent portions of animal- vs. plant-based Protein Foods, as defined by the Dietary Guidelines for Americans on essential amino acids bioavailability in young and older adults: crossover randomized controlled trials. Gavin Connolly

**Insulin**								
**Pork**	14.0 ± 2.4[a]	35.2 ± 2.3[a]	31.1 ± 2.3[abc]	20.9 ± 2.3[a]	14.8 ± 2.3[a]	12.1 ± 2.3[a]	9.3 ± 2.3[a]	2139 ± 232[a]
**Egg**	10.3 ± 1.8[a]	33.3 ± 1.8[a]	25.0 ± 1.8[ab]	15.2 ± 1.8[a]	12.6 ± 1.8[a]	8.2 ± 1.8[a]	7.2 ± 1.8[a]	1946 ± 237[a]
**Black beans**	12.1 ± 2.0[a]	34.5 ± 2.0[a]	31.0 ± 2.0[ac]	18.6 ± 2.0[a]	14.5 ± 2.0[a]	10.5 ± 2.0[a]	9.3 ± 2.0[a]	2181 ± 232[a]
**Almonds**	11.8 ± 1.9[a]	32.7 ± 1.9[a]	25.3 ± 1.9[abc]	15.9 ± 1.9[a]	10.7 ± 1.9[a]	10.5 ± 1.9[a]	9.3 ± 1.9[a]	1892 ± 232[a]

[1] Values are least-squares means ± SE; n = 30 (15 females, 15 males). Plasma amino acids, glucose, and insulin concentrations were measured over 300 minutes. Data were analyzed using a doubly repeated measures ANOVA (proc mixed; SAS version 9.3). Differences in means were determined using a Bonferroni post hoc test. Trial-by-time interactions for TAAs, EAAs, BCAAs, leucine, and NEAAs were all significant, Tukey adjusted $P < .001$. TAA, total amino acids (sum of Ala, Arg, Apn, Asp, Cys, Gln, Glu, Gly, His, Ile, Leu, Lys, Met, Phe, Pro, Ser, Thr, Trp, Tyr, and Val); EAAs, essential amino acids (sum of His, Ile, Leu, Lys, Met, Phe, Thr, Trp, and Val); BCAAs, branched-chain amino acids (sum of Leu, Ile, and Val); NEAA, non-essential amino acids (sum of Ala, Arg, Asn, Asp, Cys, Gln, Glu, Gly, Pro, Ser, and Tyr); iAUCpos, positive incremental area under the curve.

[2] Values are least-squares means ± SE. Plasma amino acids, glucose, and insulin iAUCpos are reported over the 300-minute postprandial period with 0 minute time point used as baseline. Data were analyzed using a repeated measures ANOVA (proc mixed; SAS version 9.3) and differences in means were determined using a Bonferroni post hoc test, Bonferroni adjusted $P < 0.001$.

[3] Different superscript letters are significantly different from each other within time point, Bonferroni adjusted $P < 0.05$; n = 30 (15 females, 15 males).

Effects of consuming ounce equivalent portions of animal- vs. plant-based Protein Foods, as defined by the Dietary Guidelines for Americans on essential amino acids bioavailability in young and older adults: crossover randomized controlled trials. Gavin Connolly

Table S4. Fasting and postprandial amino acids, glucose, and insulin concentrations and positive incremental areas under the curve for older adults.

	Time (mins)							iAUCpos
	**0**	**30**	**60**	**120**	**180**	**240**	**300**	
				µg/mL[1]				µg/mL*300 min[2]
**TAAs**								
**Pork**	386 ± 9^{a}	419 ± 9^{a}	437 ± 9^{abc}	482 ± 9^{a}	459 ± 9^{a}	421 ± 9^{ab}	398 ± 9^{a}	16314 ± 1377^{a}
**Egg**	389 ± 9^{a}	411 ± 9^{a}	443 ± 9^{ab}	450 ± 9^{b}	437 ± 9^{a}	411 ± 9^{abc}	390 ± 9^{a}	11113 ± 1394^{b}
**Black beans**	388 ± 8^{a}	403 ± 8^{a}	413 ± 8^{ac}	413 ± 8^{c}	400 ± 8^{b}	389 ± 8^{ac}	385 ± 8^{a}	6786 ± 1381^{c}
**Almonds**	386 ± 9^{a}	411 ± 9^{a}	419 ± 9^{abc}	405 ± 9^{c}	394 ± 9^{b}	394 ± 9^{abc}	380 ± 9^{a}	6241 ± 1364^{c}
**EAAs**								
**Pork**	110 ± 4^{a}	121 ± 4^{a}	131 ± 4^{a}	154 ± 4^{a}	151 ± 4^{a}	136 ± 4^{a}	127 ± 4^{a}	8424 ± 496^{a}
**Egg**	113 ± 4^{a}	121 ± 4^{a}	133 ± 4^{a}	141 ± 4^{b}	140 ± 4^{a}	131 ± 4^{a}	123 ± 4^{ab}	5725 ± 502^{b}
**Black beans**	111 ± 2^{a}	116 ± 2^{a}	117 ± 2^{b}	116 ± 2^{c}	115 ± 2^{b}	113 ± 2^{b}	113 ± 2^{bc}	2084 ± 497^{c}
**Almonds**	112 ± 3^{a}	119 ± 3^{a}	119 ± 3^{b}	115 ± 3^{c}	111 ± 3^{b}	113 ± 3^{b}	111 ± 3^{c}	1368 ± 492^{c}
**BCAAs**								
**Pork**	44 ± 2^{a}	49 ± 2^{a}	53 ± 2^{a}	63 ± 2^{a}	65 ± 2^{a}	59 ± 2^{a}	56 ± 2^{a}	4268 ± 256^{a}
**Egg**	46 ± 2^{a}	49 ± 2^{a}	54 ± 2^{a}	59 ± 2^{b}	61 ± 2^{a}	57 ± 2^{a}	55 ± 2^{a}	3145 ± 259^{b}
**Black beans**	45 ± 1^{a}	47 ± 1^{a}	46 ± 1^{b}	46 ± 1^{c}	46 ± 1^{b}	47 ± 1^{b}	48 ± 1^{b}	866 ± 256^{c}
**Almonds**	45 ± 1^{a}	48 ± 1^{a}	47 ± 1^{b}	45 ± 1^{c}	45 ± 1^{b}	47 ± 1^{b}	47 ± 1^{b}	675 ± 254^{c}
**Leucine**								
**Pork**	14 ± 1^{a}	16 ± 1^{a}	17 ± 1^{a}	21 ± 1^{a}	21 ± 1^{a}	19 ± 1^{a}	18 ± 1^{a}	1462 ± 88^{a}
**Egg**	14 ± 1^{a}	16 ± 1^{a}	17 ± 1^{a}	19 ± 1^{b}	19 ± 1^{b}	18 ± 1^{a}	17 ± 1^{ab}	972 ± 89^{b}
**Black beans**	14 ± 1^{a}	15 ± 1^{a}	15 ± 1^{b}	14 ± 1^{c}	14 ± 1^{c}	15 ± 1^{b}	15 ± 1^{bcd}	284 ± 88^{c}
**Almonds**	14 ± 1^{a}	16 ± 1^{a}	15 ± 1^{b}	14 ± 1^{c}	14 ± 1^{c}	15 ± 1^{b}	15 ± 1^{cd}	195 ± 87^{c}
				mg/dL				mg/dL*300 min
**Glucose**								
**Pork**	76 ± 2^{a}	88 ± 2^{a}	83 ± 2^{a}	77 ± 2^{a}	81 ± 2^{a}	82 ± 2^{a}	83 ± 2^{a}	2001 ± 263^{a}
**Egg**	79 ± 3^{a}	87 ± 3^{abc}	84 ± 3^{a}	78 ± 3^{a}	80 ± 3^{a}	83 ± 3^{a}	85 ± 3^{a}	1609 ± 266^{a}
**Black beans**	78 ± 3^{a}	90 ± 3^{bc}	89 ± 3^{a}	79 ± 3^{a}	79 ± 3^{a}	80 ± 3^{a}	82 ± 3^{a}	2091 ± 263^{a}
**Almonds**	79 ± 2^{a}	88 ± 2^{abc}	84 ± 2^{a}	80 ± 2^{a}	79 ± 2^{a}	81 ± 2^{a}	82 ± 2^{a}	1472 ± 261^{a}
				µIU/mL				µIU/mL*300 min

Effects of consuming ounce equivalent portions of animal- vs. plant-based Protein Foods, as defined by the Dietary Guidelines for Americans on essential amino acids bioavailability in young and older adults: crossover randomized controlled trials. Gavin Connolly

**Insulin**								
**Pork**	8.3 ± 1.5[a]	24.1 ± 1.5[a]	26.7 ± 1.5[abc]	13.6 ± 1.5[a]	10.1 ± 1.5[a]	6.4 ± 1.5[a]	6.0 ± 1.5[a]	1819 ± 236[a]
**Egg**	9.4 ± 1.4[a]	22.8 ± 1.4[a]	23.5 ± 1.4[abd]	13.8 ± 1.4[a]	10.3 ± 1.4[a]	8.3 ± 1.4[a]	7.5 ± 1.4[a]	1530 ± 239[a]
**Black beans**	8.1 ± 1.8[a]	24.6 ± 1.8[a]	29.4 ± 1.8[c]	13.6 ± 2.0[a]	8.6 ± 1.8[a]	7.0 ± 1.8[a]	6.5 ± 1.8[a]	1930 ± 237[a]
**Almonds**	8.3 ± 1.5[a]	25.8 ± 1.5[a]	21.7 ± 1.5[d]	10.6 ± 1.5[a]	7.6 ± 1.5[a]	6.4 ± 1.5[a]	6.3 ± 1.5[a]	1301 ± 236[a]

[1] Values are least-squares means ± SE; n = 25 (15 females, 10 males). Plasma amino acids, glucose, and insulin concentrations were measured over 300 minutes. Data were analyzed using a doubly repeated measures ANOVA (proc mixed; SAS version 9.3). Differences in means were determined using a Bonferroni post hoc test. Trial-by-time interactions for TAAs, EAAs, BCAAs, leucine, and NEAAs were all significant, Tukey adjusted $P < .001$. TAA, total amino acids (sum of Ala, Arg, Apn, Asp, Cys, Gln, Glu, Gly, His, Ile, Leu, Lys, Met, Phe, Pro, Ser, Thr, Trp, Tyr, and Val); EAAs, essential amino acids (sum of His, Ile, Leu, Lys, Met, Phe, Thr, Trp, and Val); BCAAs, branched-chain amino acids (sum of Leu, Ile, and Val); NEAA, non-essential amino acids (sum of Ala, Arg, Asn, Asp, Cys, Gln, Glu, Gly, Pro, Ser, and Tyr); iAUCpos, positive incremental area under the curve.

[2] Values are least-squares means ± SE. Plasma amino acids, glucose, and insulin iAUCpos are reported over the 300-minute postprandial period with 0 minute time point used as baseline. Data were analyzed using a repeated measures ANOVA (proc mixed; SAS version 9.3) and differences in means were determined using a Bonferroni post hoc test, Bonferroni adjusted $P < 0.05$.

[3] Different superscript letters are significantly different from each other within time point, Bonferroni adjusted $P < 0.05$; n = 25 (15 females, 10 males).

Effects of consuming ounce equivalent portions of animal- vs. plant-based Protein Foods, as defined by the Dietary Guidelines for Americans on essential amino acids bioavailability in young and older adults: crossover randomized controlled trials. Gavin Connolly

Table S5. Fasting and postprandial amino acids, glucose, and insulin concentrations and positive incremental areas under the curve for young and older adults combined.

	Time (mins)							iAUCpos
	**0**	**30**	**60**	**120**	**180**	**240**	**300**	
				µg/mL[1]				µg/mL*300 min[2]
**TAAs**								
**Pork**	384 ± 7^{a}	415 ± 7^{a}	436 ± 7^{a}	476 ± 7^{a}	449 ± 7^{a}	411 ± 7^{a}	388 ± 7^{a}	15508 ± 803^{a}
**Egg**	384 ± 6^{a}	408 ± 6^{abc}	434 ± 6^{a}	444 ± 6^{b}	430 ± 6^{b}	400 ± 6^{ab}	380 ± 6^{a}	10603 ± 820^{b}
**Black beans**	378 ± 5^{a}	397 ± 5^{bc}	404 ± 5^{b}	404 ± 5^{c}	394 ± 5^{c}	382 ± 5^{bc}	374 ± 5^{a}	6463 ± 806^{c}
**Almonds**	385 ± 6^{a}	410 ± 6^{abc}	412 ± 6^{b}	396 ± 6^{c}	390 ± 6^{c}	382 ± 6^{bc}	374 ± 6^{a}	5252 ± 803^{c}
**EAAs**								
**Pork**	120 ± 2^{a}	131 ± 2^{a}	142 ± 2^{a}	163 ± 2^{a}	156 ± 2^{a}	140 ± 2^{a}	131 ± 2^{a}	7900 ± 313^{a}
**Egg**	122 ± 2^{a}	130 ± 2^{a}	140 ± 2^{a}	149 ± 2^{b}	147 ± 2^{b}	134 ± 2^{a}	127 ± 2^{a}	5252 ± 320^{b}
**Black beans**	117 ± 2^{a}	123 ± 2^{b}	125 ± 2^{b}	124 ± 2^{c}	123 ± 2^{c}	120 ± 2^{b}	117 ± 2^{b}	2015 ± 314^{c}
**Almonds**	121 ± 2^{a}	128 ± 2^{ab}	126 ± 2^{b}	120 ± 2^{c}	119 ± 2^{c}	118 ± 2^{b}	116 ± 2^{b}	1290 ± 313^{c}
**BCAAs**								
**Pork**	50 ± 1^{a}	55 ± 1^{a}	58 ± 1^{a}	69 ± 1^{a}	68 ± 1^{a}	62 ± 1^{a}	58 ± 1^{a}	3806 ± 158^{a}
**Egg**	51 ± 1^{a}	55 ± 1^{a}	59 ± 1^{a}	64 ± 1^{b}	65 ± 1^{a}	60 ± 1^{a}	57 ± 1^{a}	2959 ± 162^{b}
**Black beans**	50 ± 1^{a}	52 ± 1^{a}	51 ± 1^{b}	51 ± 1^{c}	51 ± 1^{b}	51 ± 1^{b}	50 ± 1^{b}	775 ± 159^{c}
**Almonds**	51 ± 1^{a}	54 ± 1^{a}	52 ± 1^{b}	49 ± 1^{c}	50 ± 1^{b}	51 ± 1^{b}	50 ± 1^{b}	627 ± 158^{c}
**Leucine**								
**Pork**	16 ± 1^{a}	18 ± 1^{a}	19 ± 1^{a}	23 ± 1^{a}	22 ± 1^{a}	19 ± 1^{a}	18 ± 1^{a}	1295 ± 52^{a}
**Egg**	16 ± 1^{a}	17 ± 1^{a}	19 ± 1^{a}	20 ± 1^{b}	20 ± 1^{b}	18 ± 1^{b}	17 ± 1^{b}	868 ± 54^{b}
**Black beans**	16 ± 1^{a}	17 ± 1^{a}	16 ± 1^{b}	16 ± 1^{c}	16 ± 1^{c}	16 ± 1^{c}	16 ± 1^{c}	235 ± 53^{c}
**Almonds**	16 ± 1^{a}	17 ± 1^{a}	16 ± 1^{b}	15 ± 1^{c}	15 ± 1^{c}	16 ± 1^{c}	16 ± 1^{c}	170 ± 53^{c}
				mg/dL				mg/dL*300 min
**Glucose**								
**Pork**	78 ± 1^{a}	88 ± 1^{a}	81 ± 1^{a}	78 ± 1^{a}	80 ± 1^{a}	82 ± 1^{a}	82 ± 1^{a}	1668 ± 148^{a}
**Egg**	78 ± 1^{a}	88 ± 1^{a}	80 ± 1^{a}	77 ± 1^{a}	79 ± 1^{a}	81 ± 1^{a}	82 ± 1^{a}	1395 ± 151^{a}
**Black beans**	80 ± 1^{a}	91 ± 1^{a}	86 ± 1^{b}	79 ± 1^{a}	79 ± 1^{a}	81 ± 1^{a}	82 ± 1^{a}	1557 ± 149^{a}
**Almonds**	80 ± 1^{a}	88 ± 1^{a}	82 ± 1^{ab}	80 ± 1^{a}	79 ± 1^{a}	81 ± 1^{a}	82 ± 1^{a}	1164 ± 148^{a}
				µIU/mL				µIU/mL*300 min

**Insulin**								
**Pork**	11.3 ± 1.5[a]	29.8 ± 1.5[a]	29.1 ± 1.5[a]	16.4 ± 1.5[a]	12.6 ± 1.5[a]	9.4 ± 1.5[a]	7.8 ± 1.5[a]	1999 ± 169[a]
**Egg**	9.7 ± 1.2[a]	27.6 ± 1.2[a]	24.2 ± 1.2[b]	14.4 ± 1.2[a]	11.4 ± 1.2[a]	8.8 ± 1.2[a]	7.2 ± 1.2[a]	1749 ± 172[a]
**Black beans**	10.1 ± 1.4[a]	29.2 ± 1.4[a]	30.3 ± 1.4[a]	16.2 ± 1.4[a]	11.2 ± 1.4[a]	8.8 ± 1.4[a]	8.1 ± 1.4[a]	2062 ± 170[a]
**Almonds**	10.0 ± 1.2[a]	29.2 ± 1.2[a]	23.5 ± 1.2[b]	13.2 ± 1.2[a]	9.3 ± 1.2[a]	8.4 ± 1.2[a]	7.7 ± 1.2[a]	1588 ± 170[a]

[1] Values are least-squares means ± SE; n = 55 (30 females, 25 males). Plasma amino acids, glucose, and insulin concentrations were measured over 300 minutes. Data were analyzed using a doubly repeated measures ANOVA (proc mixed; SAS version 9.3). Differences in means were determined using a Bonferroni post hoc test. Trial-by-time interactions for TAAs, EAAs, BCAAs, leucine, and NEAAs were all significant, Tukey adjusted $P < .001$. TAA, total amino acids (sum of Ala, Arg, Apn, Asp, Cys, Gln, Glu, Gly, His, Ile, Leu, Lys, Met, Phe, Pro, Ser, Thr, Trp, Tyr, and Val); EAAs, essential amino acids (sum of His, Ile, Leu, Lys, Met, Phe, Thr, Trp, and Val); BCAAs, branched-chain amino acids (sum of Leu, Ile, and Val); NEAA, non-essential amino acids (sum of Ala, Arg, Asn, Asp, Cys, Gln, Glu, Gly, Pro, Ser, and Tyr); iAUCpos, positive incremental area under the curve.

[2] Values are least-squares means ± SE. Plasma amino acids, glucose, and insulin iAUCpos are reported over the 300-minute postprandial period with 0 minute time point used as baseline. Data were analyzed using a repeated measures ANOVA (proc mixed; SAS version 9.3) and differences in means were determined using a Bonferroni post hoc test, Bonferroni adjusted $P < 0.05$.

[3] Different superscript letters are significantly different from each other within time point, Bonferroni adjusted $P < 0.05$; n = 55 (30 females, 25 males).

Effects of consuming ounce equivalent portions of animal- vs. plant-based Protein Foods, as defined by the Dietary Guidelines for Americans on essential amino acids bioavailability in young and older adults: crossover randomized controlled trials. Gavin Connolly

Table S6. Fasting and postprandial amino acids, glucose, and insulin concentrations and positive incremental areas under the curve for females.

	Time (mins)							iAUCpos
	**0**	**30**	**60**	**120**	**180**	**240**	**300**	
				μg/mL[1]				μg/mL*300 min[2]
**TAAs**								
**Pork**	372 ± 9^{a}	404 ± 9^{a}	421 ± 9^{abc}	489 ± 9^{a}	448 ± 9^{a}	412 ± 9^{a}	384 ± 9^{a}	18070 ± 1078^{a}
**Egg**	377 ± 8^{a}	402 ± 8^{a}	422 ± 8^{b}	441 ± 8^{b}	428 ± 8^{a}	403 ± 8^{ab}	374 ± 8^{a}	11310 ± 1044^{b}
**Black beans**	364 ± 7^{a}	391 ± 7^{a}	395 ± 7^{c}	402 ± 7^{c}	389 ± 7^{b}	379 ± 7^{b}	366 ± 7^{a}	8047 ± 1062^{bc}
**Almonds**	374 ± 8^{a}	404 ± 8^{a}	406 ± 8^{abc}	399 ± 8^{c}	385 ± 8^{b}	379 ± 8^{b}	367 ± 8^{a}	6656 ± 1096^{c}
**EAAs**								
**Pork**	112 ± 3^{a}	123 ± 3^{a}	132 ± 3^{a}	163 ± 3^{a}	158 ± 3^{a}	143 ± 3^{a}	129 ± 3^{a}	7346 ± 401^{a}
**Egg**	114 ± 3^{a}	123 ± 3^{a}	130 ± 3^{a}	144 ± 3^{b}	144 ± 3^{b}	133 ± 3^{a}	122 ± 3^{a}	4689 ± 413^{b}
**Black beans**	109 ± 2^{a}	117 ± 2^{a}	118 ± 2^{b}	119 ± 2^{c}	119 ± 2^{c}	116 ± 2^{b}	112 ± 2^{b}	2023 ± 402^{c}
**Almonds**	112 ± 2^{a}	120 ± 2^{a}	118 ± 2^{b}	116 ± 2^{c}	115 ± 2^{c}	115 ± 2^{b}	111 ± 2^{b}	1184 ± 401^{c}
**BCAAs**								
**Pork**	45 ± 2^{a}	50 ± 2^{a}	52 ± 2^{a}	67 ± 2^{a}	68 ± 2^{a}	62 ± 2^{a}	57 ± 2^{a}	4488 ± 208^{a}
**Egg**	46 ± 2^{a}	49 ± 2^{a}	52 ± 2^{a}	59 ± 2^{b}	62 ± 2^{b}	58 ± 2^{a}	54 ± 2^{a}	3166 ± 206^{b}
**Black beans**	43 ± 1^{a}	46 ± 1^{a}	46 ± 1^{b}	47 ± 1^{c}	48 ± 1^{c}	47 ± 1^{b}	43 ± 1^{b}	1107 ± 207^{c}
**Almonds**	44 ± 1^{a}	48 ± 1^{a}	46 ± 1^{b}	45 ± 1^{c}	45 ± 1^{c}	47 ± 1^{b}	46 ± 1^{b}	898 ± 213^{c}
**Leucine**								
**Pork**	14 ± 1^{a}	16 ± 1^{a}	17 ± 1^{a}	22 ± 1^{a}	22 ± 1^{a}	19 ± 1^{a}	18 ± 1^{a}	1514 ± 68^{a}
**Egg**	14 ± 1^{a}	16 ± 1^{a}	16 ± 1^{a}	18 ± 1^{b}	19 ± 1^{b}	17 ± 1^{b}	16 ± 1^{b}	920 ± 68^{b}
**Black beans**	14 ± 1^{a}	15 ± 1^{a}	15 ± 1^{b}	14 ± 1^{c}	15 ± 1^{c}	15 ± 1^{c}	15 ± 1^{b}	332 ± 68^{c}
**Almonds**	14 ± 1^{a}	15 ± 1^{a}	14 ± 1^{b}	13 ± 1^{c}	14 ± 1^{c}	15 ± 1^{c}	14 ± 1^{b}	236 ± 70^{c}
				mg/dL				mg/dL*300 min
**Glucose**								
**Pork**	77 ± 2^{a}	86 ± 2^{a}	81 ± 2^{a}	77 ± 2^{a}	80 ± 2^{a}	81 ± 2^{a}	82 ± 2^{a}	1651 ± 195^{a}
**Egg**	78 ± 2^{a}	86 ± 2^{a}	77 ± 2^{a}	77 ± 2^{a}	79 ± 2^{a}	82 ± 2^{a}	82 ± 2^{a}	1462 ± 193^{a}
**Black beans**	79 ± 2^{a}	89 ± 2^{a}	84 ± 2^{a}	79 ± 2^{a}	79 ± 2^{a}	81 ± 2^{a}	82 ± 2^{a}	1594 ± 193^{a}
**Almonds**	78 ± 2^{a}	86 ± 2^{a}	80 ± 2^{a}	78 ± 2^{a}	79 ± 2^{a}	82 ± 2^{a}	82 ± 2^{a}	1229 ± 199^{a}
				μIU/mL				μIU/mL*300 min

Effects of consuming ounce equivalent portions of animal- vs. plant-based Protein Foods, as defined by the Dietary Guidelines for Americans on essential amino acids bioavailability in young and older adults: crossover randomized controlled trials. Gavin Connolly

**Insulin**								
**Pork**	10.0 ± 2.0[a]	28.6 ± 2.0[a]	26.6 ± 2.0[abc]	14.7 ± 2.0[a]	12.0 ± 2.0[a]	10.2 ± 2.0[a]	7.3 ± 2.0[a]	2061 ± 224[a]
**Egg**	8.8 ± 1.6[a]	25.7 ± 1.6[a]	22.6 ± 1.6[ab]	14.1 ± 1.6[a]	12.1 ± 1.6[a]	9.0 ± 1.6[a]	7.1 ± 1.6[a]	1780 ± 223[a]
**Black beans**	8.9 ± 1.9[a]	28.4 ± 1.9[a]	28.7 ± 1.9[c]	15.1 ± 1.9[a]	10.0 ± 1.9[a]	7.8 ± 1.9[a]	7.1 ± 1.9[a]	2045 ± 223[a]
**Almonds**	9.0 ± 1.7[a]	27.5 ± 1.7[a]	19.9 ± 1.7[d]	10.6 ± 1.7[a]	8.5 ± 1.7[a]	7.4 ± 1.7[a]	7.0 ± 1.7[a]	1376 ± 227[a]

[1] Values are least-squares means ± SE; n = 30 females. Plasma amino acids, glucose, and insulin concentrations were measured over 300 minutes. Data were analyzed using a doubly repeated measures ANOVA (proc mixed; SAS version 9.3). Differences in means were determined using a Bonferroni post hoc test. Trial-by-time interactions for TAAs, EAAs, BCAAs, leucine, and NEAAs were all significant, Tukey adjusted $P < .001$. TAA, total amino acids (sum of Ala, Arg, Apn, Asp, Cys, Gln, Glu, Gly, His, Ile, Leu, Lys, Met, Phe, Pro, Ser, Thr, Trp, Tyr, and Val); EAAs, essential amino acids (sum of His, Ile, Leu, Lys, Met, Phe, Thr, Trp, and Val); BCAAs, branched-chain amino acids (sum of Leu, Ile, and Val); NEAA, non-essential amino acids (sum of Ala, Arg, Asn, Asp, Cys, Gln, Glu, Gly, Pro, Ser, and Tyr); iAUCpos, positive incremental area under the curve.

[2] Values are least-squares means ± SE. Plasma amino acids, glucose, and insulin iAUCpos are reported over the 300-minute postprandial period with 0 minute time point used as baseline. Data were analyzed using a repeated measures ANOVA (proc mixed; SAS version 9.3) and differences in means were determined using a Bonferroni post hoc test, Bonferroni adjusted $P < 0.05$.

[3] Different superscript letters are significantly different from each other within time point, Bonferroni adjusted $P < 0.05$; n = 30).

Effects of consuming ounce equivalent portions of animal- vs. plant-based Protein Foods, as defined by the Dietary Guidelines for Americans on essential amino acids bioavailability in young and older adults: crossover randomized controlled trials. Gavin Connolly

Table S7. Fasting and postprandial amino acids, glucose, and insulin concentrations and positive incremental areas under the curve for males.

	Time (mins)							iAUCpos
	**0**	**30**	**60**	**120**	**180**	**240**	**300**	
				µg/mL[1]				µg/mL*300 min[2]
**TAAs**								
**Pork**	396 ± 10[a]	426 ± 10[a]	451 ± 10[a]	473 ± 10[a]	450 ± 10[a]	410 ± 10[a]	391 ± 10[a]	12947 ± 1155[a]
**Egg**	390 ± 9[a]	413 ± 9[a]	445 ± 9[a]	447 ± 9[a]	431 ± 9[a]	396 ± 9[a]	385 ± 9[a]	9976 ± 1235[a]
**Black beans**	392 ± 8[a]	402 ± 8[a]	413 ± 8[b]	406 ± 8[b]	399 ± 8[b]	386 ± 8[a]	381 ± 8[a]	4879 ± 1169[b]
**Almonds**	397 ± 8[a]	417 ± 8[a]	417 ± 8[ab]	399 ± 8[b]	385 ± 8[b]	384 ± 8[a]	367 ± 8[a]	3848 ± 1152[b]
**EAAs**								
**Pork**	127 ± 4[a]	140 ± 4[a]	151 ± 4[a]	163 ± 4[a]	155 ± 4[a]	138 ± 4[a]	132 ± 4[a]	6603 ± 455[a]
**Egg**	129 ± 3[a]	137 ± 3[a]	149 ± 3[a]	155 ± 3[a]	150 ± 3[a]	134 ± 3[a]	131 ± 3[a]	4692 ± 480[a]
**Black beans**	126 ± 2[a]	130 ± 2[a]	131 ± 2[b]	129 ± 2[b]	126 ± 2[b]	122 ± 2[b]	122 ± 2[a]	1213 ± 460[b]
**Almonds**	129 ± 2[a]	135 ± 2[a]	133 ± 2[b]	126 ± 2[b]	124 ± 2[b]	122 ± 2[b]	122 ± 2[a]	787 ± 455[b]
**BCAAs**								
**Pork**	55 ± 2[a]	60 ± 2[a]	65 ± 2[a]	71 ± 2[a]	69 ± 2[a]	61 ± 2[a]	59 ± 2[a]	3123 ± 231[a]
**Egg**	56 ± 2[a]	60 ± 2[a]	65 ± 2[a]	70 ± 2[a]	68 ± 2[a]	61 ± 2[a]	60 ± 2[a]	2752 ± 244[a]
**Black beans**	56 ± 1[a]	57 ± 1[a]	57 ± 1[b]	56 ± 1[b]	55 ± 1[b]	54 ± 1[b]	53 ± 1[b]	443 ± 234[b]
**Almonds**	57 ± 1[a]	60 ± 1[a]	59 ± 1[b]	54 ± 1[b]	54 ± 1[b]	54 ± 1[b]	54 ± 1[b]	355 ± 231[b]
**Leucine**								
**Pork**	18 ± 1[a]	20 ± 1[a]	21 ± 1[a]	23 ± 1[a]	22 ± 1[a]	19 ± 1[a]	19 ± 1[a]	1075 ± 76[a]
**Egg**	18 ± 1[a]	19 ± 1[a]	21 ± 1[a]	22 ± 1[a]	21 ± 1[a]	19 ± 1[ab]	18 ± 1[ab]	816 ± 80[a]
**Black beans**	18 ± 1[a]	18 ± 1[a]	18 ± 1[b]	17 ± 1[b]	17 ± 1[b]	17 ± 1[b]	16 ± 1[b]	138 ± 77[b]
**Almonds**	18 ± 1[a]	20 ± 1[a]	19 ± 1[b]	17 ± 1[b]	17 ± 1[b]	17 ± 1[b]	17 ± 1[ab]	104 ± 76[b]
				mg/dL				mg/dL*300 min
**Glucose**								
**Pork**	78 ± 2[a]	90 ± 2[a]	81 ± 2[a]	78 ± 2[a]	81 ± 2[a]	83 ± 2[a]	83 ± 2[a]	1685 ± 216[a]
**Egg**	79 ± 2[a]	90 ± 2[a]	83 ± 2[a]	77 ± 2[a]	79 ± 2[a]	81 ± 1[a]	82 ± 2[a]	1462 ± 229[a]
**Black beans**	81 ± 2[a]	86± 2[a]	80 ± 2[a]	78 ± 2[a]	79 ± 2[a]	82 ± 2[a]	82 ± 2[a]	1594 ± 219[a]
**Almonds**	81 ± 2[a]	89 ± 2[a]	84 ± 2[a]	81 ± 2[a]	79 ± 2[a]	81 ± 2[a]	82 ± 2[a]	1098 ± 216[a]
				µIU/mL				µIU/mL*300 min

**Insulin**								
**Pork**	12.7 ± 2.2[a]	31.1 ± 2.2[a]	31.6 ± 2.2[abc]	18.9 ± 2.2[a]	13.2 ± 2.2[a]	8.6 ± 2.2[a]	8.3 ± 2.2[a]	1936 ± 250[a]
**Egg**	10.8 ± 1.8[a]	29.6 ± 1.8[a]	25.9 ± 1.8[ab]	14.8 ± 1.8[a]	10.6 ± 1.8[a]	7.3 ± 1.8[a]	7.4 ± 1.8[a]	1717 ± 259[a]
**Black beans**	11.4 ± 2.1[a]	30.1 ± 2.1[a]	31.9 ± 2.1[ac]	17.2 ± 2.1[a]	12.5 ± 2.1[a]	9.8 ± 2.1[a]	8.9 ± 2.1[a]	2078 ± 251[a]
**Almonds**	11.1 ± 1.8[a]	30.9 ± 1.8[a]	27.0 ± 1.8[abc]	15.9 ± 1.8[a]	10.1 ± 1.8[a]	9.5 ± 1.8[a]	8.5 ± 1.8[a]	1799 ± 249[a]

[1] Values are least-squares means ± SE; n = 25 males. Plasma amino acids, glucose, and insulin concentrations were measured over 300 minutes. Data were analyzed using a doubly repeated measures ANOVA (proc mixed; SAS version 9.3). Differences in means were determined using a Bonferroni post hoc test. Trial-by-time interactions for TAAs, EAAs, BCAAs, leucine, and NEAAs were all significant, Tukey adjusted $P < .001$. TAA, total amino acids (sum of Ala, Arg, Apn, Asp, Cys, Gln, Glu, Gly, His, Ile, Leu, Lys, Met, Phe, Pro, Ser, Thr, Trp, Tyr, and Val); EAAs, essential amino acids (sum of His, Ile, Leu, Lys, Met, Phe, Thr, Trp, and Val); BCAAs, branched-chain amino acids (sum of Leu, Ile, and Val); NEAA, non-essential amino acids (sum of Ala, Arg, Asn, Asp, Cys, Gln, Glu, Gly, Pro, Ser, and Tyr); iAUCpos, positive incremental area under the curve.

[2] Values are least-squares means ± SE. Plasma amino acids, glucose, and insulin iAUCpos are reported over the 300-minute postprandial period with 0 minute time point used as baseline. Data were analyzed using a repeated measures ANOVA (proc mixed; SAS version 9.3) and differences in means were determined using a Bonferroni post hoc test, Bonferroni adjusted $P < 0.05$.

[3] Different superscript letters are significantly different from each other within time point, Bonferroni adjusted $P < 0.05$; n = 25.
